# Cost-effectiveness of organised versus opportunistic mammography breast cancer screening in low-resourced country

**DOI:** 10.1136/bmjopen-2025-108486

**Published:** 2026-08-17

**Authors:** Abeer Al Rabayah, Olena Mandrik, Asem Mansour, Reem Al-Ajlouni, Tala Haddad, Hikmat Abdel-Razeq, Omar Shamieh, Kalipso Chalkidou, Richard Sullivan, Adrian Gheorghe

**Affiliations:** 1 Department of Pharmacy, Center for Drug Policy and Technology Assessment, King Hussein Cancer Center, Amman, Jordan; 2 Sheffield Centre for Health and Related Research, Population Health, The University of Sheffield School of Medicine and Population Health, Sheffield, UK; 3 King Hussein Cancer Center, Amman, Jordan; 4 Jordan Breast Cancer Program, King Hussein Cancer Foundation, Amman, Jordan; 5 Department of Palliative Medicine, King Hussein Cancer Center, Amman, Jordan; 6 Department of Performance, Financing and Delivery, WHO, Geneva, Switzerland; 7 King’s College, Institute of Cancer Policy, Centre for Conflict & Health Research, London, UK; 8 Imperial College, London, UK

**Keywords:** Cancer, Health economics, Breast tumours

## Abstract

**Abstract:**

**Objectives:**

To assess the cost-effectiveness of expanding the current opportunistic screening strategy to an organised and feasible population-based breast cancer screening strategy from the healthcare system perspective in Jordan.

**Design:**

A probabilistic, state-transition cohort decision-analytic model was developed to estimate the expected incremental life years (LYs), quality-adjusted life years (QALYs) and costs.

**Participants:**

The model includes a hypothetical cohort of average-risk asymptomatic Jordanian women aged 40 years and above.

**Interventions:**

The base case comparison was between organised mammography with a 50% attendance rate and opportunistic mammography for asymptomatic women aged 40–70 years and older over a lifetime horizon. Multiple organised mammogram screening strategies were assessed that varied in screening interval and start and end age (strategy 1: 40–60 biennial, strategy 2: 50–65 annual, strategy 3: 50–65 biennial, strategy 4: 40–70 biennial, strategy 5: 50–70 biennial and strategy 6: 40–49 annual).

**Setting:**

This model-based economic evaluation study was conducted to reflect the context of the Jordanian healthcare system.

**Measurement:**

We identified, measured and valued resources and outcomes to populate the model. Transition probabilities were estimated from the literature, the cancer registry or through calibration. The screening test’s specificity and sensitivity were obtained from the literature. Costs and utility weights were extracted from national databases and literature. Both costs and outcomes were discounted using an annual discount rate of 3%. The model’s uncertainty was tested using deterministic and probabilistic sensitivity analysis.

**Results:**

Over a lifetime horizon, strategy 3 (50–65 biennial, 50% attendance rate) resulted in a mean discounted LYs of 22.077, a mean discounted QALYs of 16.993 and a mean discounted cost of US$994 compared with opportunistic screening, which resulted in a mean discounted LYs of 22.062, a mean discounted QALYs of 16.980 and a mean discounted cost of US$889. The incremental cost-utility ratio (ICUR) relative to opportunistic screening is US$6734 per QALY. Strategy 3 ranks first as the most cost-effective organised screening strategy for the Jordanian healthcare system, followed by strategy 4 with an ICUR of US$18 024 per QALY and strategy 6 with an ICUR of US$62 418 per QALY. The three strategies were presented on the cost-effectiveness frontier. The deterministic sensitivity analysis showed that the results were sensitive to the cost of the screening package and the annual discount rate.

**Conclusion:**

At a willingness-to-pay cost-effectiveness threshold of US$42000–56000 per QALY, strategy 3 (50–65 biennial, 50% attendance rate) and strategy 4 (40–70 biennial, 50% attendance rate) are likely to be cost-effective in Jordan. However, prior to implementation, the affordability of each strategy and the availability of required infrastructure must be carefully evaluated.

**STRENGTHS AND LIMITATIONS OF THIS STUDY**

- Using calibration to generate transition probabilities using available local data as targets.
- Building a state transition cohort model that matches the Jordanian context.
- Using national sources for resource use, costing, general population utilities and epidemiological data.
- The availability of national data on health state utilities is one of the study’s limitations.
- The fragmentation of data sources was a major limitation of the study.

## Introduction

 Breast cancer is the most commonly diagnosed cancer worldwide, with 2.3 million new cases reported annually.^1^ In 2020, breast cancer represented a quarter of all cancers in females and 16% of cancer deaths in women. In 2040, new cases of breast cancer are projected to increase by 40%, and 22% will occur in transitioning countries.^2^

Breast cancer is also a significant challenge for Jordan, classified as a lower-middle-income country (LMIC). It is the most common cancer in Jordan. It accounts for 21% of all cancer cases among both genders. In 2018, 1463 cases were registered in Jordan, bringing the cumulative total to 17 731 (1996–2018).^3^ The reported crude incidence in 2015 was 34.1 per 100,000.^4^ On the other hand, the age standardised rate (ASR) from 2011 to 2019 was 44.9 per 100 000 person-years (95% CI 33.6 to 56.8), which is higher than the pooled breast cancer ASR in the Eastern Mediterranean Region during the same period.^5^ The 5-year survival rates, categorised per stages, are 96% for stage 1, 91% for stage 2, 76% for stage 3 and 32% for stage 4.^4^

The increasing incidence of breast cancer and the prevalence of late-stage diagnoses pose significant public health challenges in Jordan, placing a considerable strain on the healthcare system. Alarmingly, 50% of breast cancer cases in Jordan are diagnosed at late stages,^6^ with around 14% of those cases presenting with metastatic disease,^4^ contributing to the high management costs estimated at approximately US$42 000 per case, excluding emergency admissions and targeted biological therapies.^7^

Breast cancer screening is an important strategy to tackle the increased burden of breast cancer. Mammography screening programmes have been established worldwide, mostly in developed, high-income countries.^8^ Evidence shows that mammography screening reduces mortality by up to 20%, and it remains the gold standard for breast cancer screening in well-developed healthcare systems.^9^ However, consensus on age and screening intervals has yet to be found. For example, most European countries recommend biennial mammograms for women aged 50–69 years. In comparison, the US recommends annual or biennial screening for women aged 40–74,^10–14^ while the UK recommends triennial mammogram screening for women aged 50–70 years.^15^

In Jordan, the national breast cancer screening guidelines recommend annual screening for women aged 40+. Those guidelines were developed under the umbrella of the Jordan Breast Cancer Programme (JBCP), established in 2007 to create an enabling environment for breast cancer early detection.^16^ The JBCP adopted a down-staging strategy that relies on early diagnosis and opportunistic screening. However, a low attendance rate is a significant challenge.^17 18^ Creating awareness, improving access and quality of available services, mobile clinics, building capacity of healthcare providers and enhancing referral pathways are the main interventions implemented in Jordan to increase the attendance rate.^17 18^ However, cost and limited advice from healthcare professionals are considered key barriers to the uptake of breast cancer mammogram screening.^17^

The healthcare system in Jordan is fragmented with multiple payers and insurance schemes. 78% of the Jordanian population is covered by a type of insurance. 44% are covered by the Ministry of Health (MoH), 27% by the Royal Medical Services (RMS), 7% by private insurance and 21% are not insured.^18^ Breast cancer screening services are not covered by private health insurance.^18^ Therefore, around 30% of the population must pay out of pocket to access mammogram screening. On the other hand, the Jordanian population who have MoH or RMS insurance have coverage of breast cancer screening services, but the attendance rate is low. Moreover, the King Hussein Cancer Centre (KHCC), a specialised comprehensive cancer centre, provides breast cancer mammogram services through the early detection clinic. In addition, the King Hussein Cancer Foundation (KHCF) launched a cancer care insurance programme that includes free coverage only for annual clinical examination.^19^ Therefore, there is a need to explore expanding the current opportunistic screening strategy into an organised mammography screening policy in Jordan.^17^

Despite the importance of having an organised breast cancer screening programme for Jordan, several uncertainties exist. First, there is no information about the cost-effectiveness of an organised breast cancer screening strategy in Jordan to inform national guidelines. Second, there is uncertainty about the starting age of screening and the screening interval that is associated with the highest value in the Jordanian context. Therefore, this study aimed to assess the cost-effectiveness of several organised mammography breast cancer screening strategies compared with the current opportunistic mammography breast cancer screening. The ultimate objective of this research is to inform breast cancer mammogram screening policies in Jordan within a fragmented healthcare system and with limited resources.

## Methods

The study is presented using the structure of the Consolidated Health Economic Evaluation Reporting Standards (CHEERs) checklist. The completed CHEERs checklist is available in online supplemental materials.

### Health economics analysis plan

**Figure 1:**
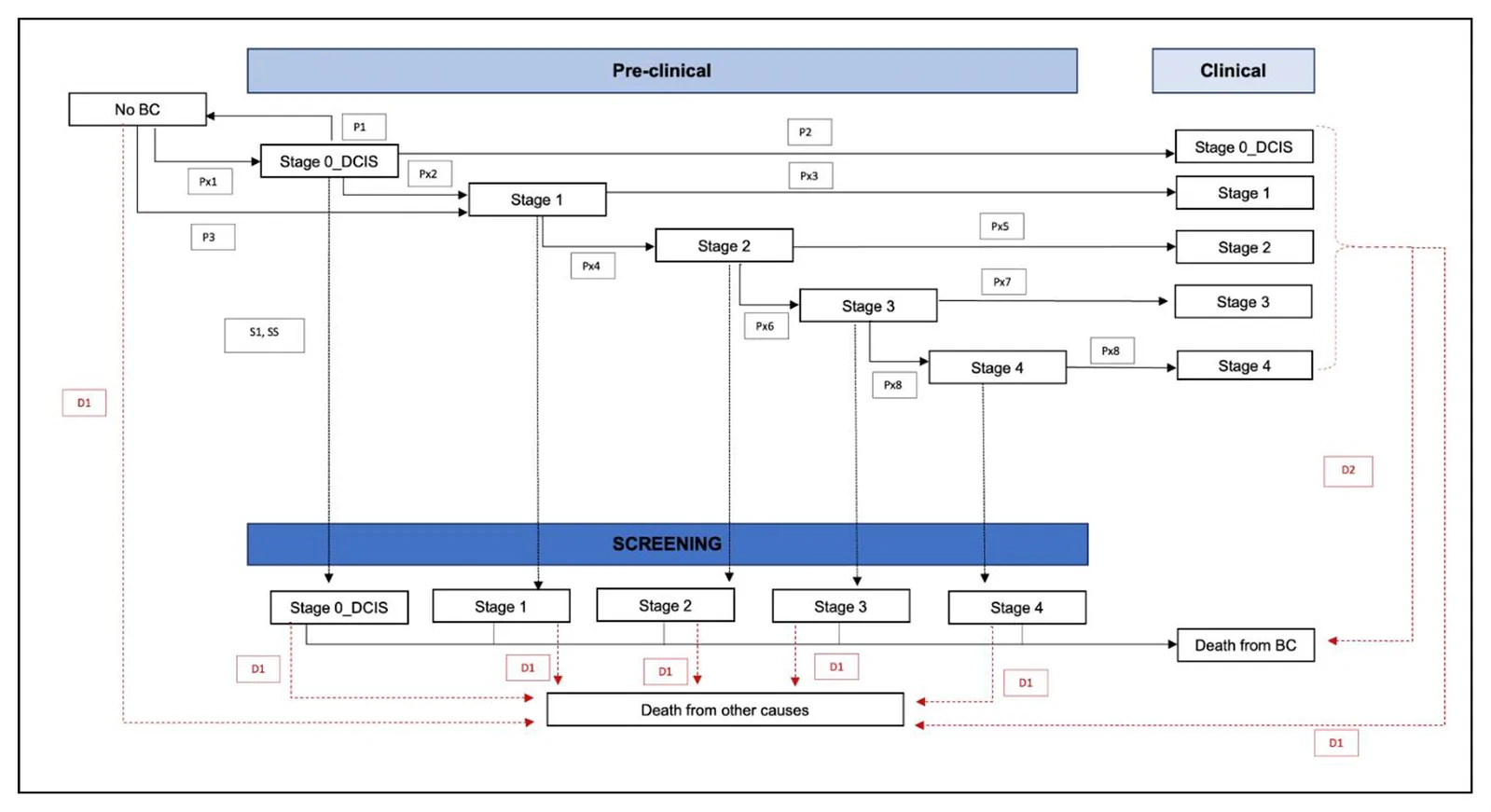
Model structure. BC, breast cancer; DCIS, ductal carcinoma in situ; D, death; P, transition probability; SS, sensitivity and specificity of mammography; S1, attendance rate. Adapted from Schiller Fruehwirth et al^22^

This is a cost-effectiveness study using a decision-analytic modelling framework as the study vehicle. This model-based full economic evaluation study was populated with aggregated, not individual-level data. It used published secondary data and real-world data, with the latter primarily collected for the study.^20 21^ We adopted the structure of a previously published model for Schiller-Fruehwirth *et al*^22^ that assessed a similar decision problem. Figure 1 shows the adopted model structure. However, we used a different modelling technique in our study. A scientific advisory group was established and consulted on the model structure, assumptions and input validation. A conceptualisation of the model was developed, documented and shared with the project’s stakeholders before its implementation. The model development process was iterative, with modifications and improvements following discussions with the project’s scientific advisory group, until the final version was obtained. Table 1 shows the project’s decision problem.

**Table 1:** Project’s decision problem

Population	Asymptomatic average risk women in Jordan above the age of 40 years
Intervention	Different strategies of organised mammography breast cancer screening
Comparator	Opportunistic breast cancer screening (annual, 40–70 years)
Outcomes	Number of breast cancer cases detected. Life years saved. Quality-adjusted life years (QALYs)
Type of analysis	Model-based economic evaluation (cohort simulation). Incremental cost per life year gained Incremental cost per QALY gained
Study type	Cost-effectiveness/cost-utility
Perspective	Jordanian healthcare system

QALYs, quality-adjusted life years.

### Study population

The model includes a hypothetical cohort of average-risk asymptomatic Jordanian women aged 40 years and above. This population represents the current eligible population for breast cancer screening in Jordan.

### Setting and location

This study was conducted in Jordan, a LMIC with a population of 11.7 million and a gross domestic product (GDP) per capita of US$4482.^23–25^ The life expectancy at birth of the Jordanian population is 75 years, and noncommunicable diseases are responsible for 54% of deaths.^26^ The healthcare system in Jordan is highly fragmented, with multiple public programmes providing healthcare with limited coordination. Cancer treatment is covered by the government of Jordan; however, the increased economic burden of cancer has led to changes in the insurance landscape, including the introduction of a non-profit cancer insurance programme that provides partial coverage of cancer treatment costs at the KHCC.^27^

### Interventions: screening strategies

Several mammography screening strategies were assessed across age intervals and screening frequencies. We assumed a 50% attendance rate for all strategies as a realistic but conservative participation target for a structured screening programme in Jordan. This selection was informed by the JBCP’s field experience through mobile mammography services, community outreach and facilitated referrals to hospitals.

Table 2 shows all proposed mammography screening strategies. Proposed screening strategies were based on recommendations from key stakeholders at KHCC and JBCP as well as the WHO’s recommendations for breast cancer screening for LMIC.^28^

**Table 2:** Mammography screening scenarios

Screening strategy	Age interval (years)	Mammography screening frequency	Attendance rate	Source
Comparator strategy	40–70	Annual	3.5%	National breast cancer screening guidelines^58^
Strategy 1	40–60	Biennial	50%	Recommended KHCC, JBCP
Strategy 2	50–65	Annual	50%	Recommended KHCC, JBCP
Strategy 3	50–65	Biennial	50%	Recommended KHCC, JBCP
Strategy 4	40–70	Biennial	50%	WHO recommendations for well-resourced countries^28^
Strategy 5	50–70	Biennial	50%	WHO recommendations for limited-resource countries^28^
Strategy 6	40–49 50–70	Annual Biennial	50%	Recommended KHCC, JBCP

Annual: once every year; biennial: once every 2 years.

JBCP, Jordan Breast Cancer Program; KHCC, King Hussein Cancer Center.

### Comparators

According to the Jordanian national breast cancer screening guidelines, the reference strategy is opportunistic annual mammography screening starting at age 40. Usually, women are referred for breast cancer screening by physicians during checks or visits for other health problems. Also, national breast cancer screening campaigns, especially in October, encourage women to do annual breast cancer screening from age 40 onwards. The attendance rate for the opportunistic strategy reflects the participation rate of the eligible women’s population.

### Study perspective

A healthcare system perspective was adopted. Therefore, only direct medical costs were included in the study.

### Time horizon and discount rate

The study adopts a lifetime horizon to capture the long-term costs and benefits. The maximum age was 100 years, and the resulting total years in the base case are 61 elapsed years. Both costs and outcomes were discounted at an annual rate of 3%, in accordance with the KHCC economic evaluation reference case.

### Identification, measurement and valuation of outcomes

#### Effectiveness

Transition probabilities were either extracted from the literature,^4 22^ country life tables or the KHCC cancer registry or derived through calibration (online supplemental table 1). The project’s scientific advisory group validated the model’s assumption and the derived transition probabilities.

Due to the lack of data on the progression of undiagnosed pre-clinical cancer, the model’s natural history component was calibrated using a random approach. Calibration allows assessment of model parameters based on the consistency between model predictions (outputs) and observed data.^29–31^ The calibration process involves comparing model outputs with selected empirical data, called calibration targets and exploring variations in the model’s parameters to identify combinations that yield the best fit between the model outputs and the empirical data (goodness of fit (GOF)).^29–31^

National age-specific incidence rates for 2018 and breast cancer mortality were identified as our current calibration target.^3^ We allowed unobservable input parameters to vary during the calibration process. We applied the least squares method as our GOF measure, aiming to minimise the GOF estimate. Several sets of input parameters that match the acceptance criteria were generated. Selection of the parameter set followed a rule to achieve predicted values of 1/SEE less than the observed values for breast cancer incidence and mortality.^29–31^ Calibrations were run until we achieved the best fit of the calibration target. The screening mammography sensitivity and specificity inputs were taken from the literature^22^ (online supplemental table 1). We assumed that the test accuracy for the first and subsequent mammographies is the same. In addition, we assumed that the accuracy of MRI and biopsy is 100%.

#### Utilities

Utility values for women without cancer were assumed to equal the general population utility score, adjusted for age and gender. General population utility scores were derived from the Jordanian EQ-5D-3L valuation study using the EQ VAS scores^32^ (online supplemental table 2). Utility index scores for each cancer stage were extracted from a systematic review published in 2022^33^ (online supplemental table 2). We assumed utility scores during the pre-clinical disease phase equalled the age- and gender-adjusted general population utility scores. Moreover, we assumed that the screening procedure has no impact on quality of life; therefore, disutility due to screening was not incorporated into our model.^34^

### Measurement and valuation of resources and costs

Expected resource use from screening appointments through the receipt of screening results was calculated using a process flowchart based on data from KHCC. 10% of screened women are expected to receive additional mammography views to confirm the results, and 25% are expected to be referred for MRI or biopsy. Jordanian direct medical cost data were derived from the KHCC databases and published literature.^35–37^ As Jordan has a fragmented healthcare system, we calculated a weighted average of screening costs, defined as the sum of mammography and ultrasound costs. The weighted average was based on the average price charged by the public sector, private sector and non-profit organisations. The average price was adjusted for the insurance percentage per entity and the probability of using the service at each entity.

The cost of programme administration per eligible woman was taken from the literature^22^ (online supplemental table 3). The mean treatment cost per stage was categorised into initial and follow-up costs. The calculated mean cost covered the reported costs of breast cancer per stage in KHCC^35^ (online supplemental table 3), the MoH,^36^ Jordan University Hospital^36^ and King Abdulla University Hospital^37^ (online supplemental table 3). All costs per stage were reported in US dollars and standardised using the consumer price index for the year 2022.

### Rationale and description of the model

A state-transition cohort Markov model was built to simulate the natural history of breast cancer over a lifelong time horizon.^20 21^ We adopted the model structure used in previous breast cancer models with extensive validation processes.^22^ However, we used a cohort rather than a patient-level simulation technique. Cohort Markov models in breast cancer screening models are the most commonly applied modelling technique.^38^ Our model cycle length was 1 year. Figure 1 shows the adopted model structure.

The model consisted of three main phases: pre-clinical, clinical and screening. The pre-clinical and clinical phases represent the natural progression of the disease. The preclinical phase refers to the period from the biological onset of the disease to the onset of clinical symptoms. The most important aspect in the pre-clinical phase is that the disease is still asymptomatic but detectable using a screening test. The clinical phase is the period with clinical symptoms of the disease, during which screening will be ineffective.^39^ Under each phase, several health states exist, as illustrated in figure 1.

A hypothetical cohort of average-risk asymptomatic women aged 40 years and above is expected to move in annual cycles over their lifetime, starting in the no-breast-cancer health state. However, the cohort simulation over a lifelong time horizon starts at age 20 because it simulates the natural history of cancer, in which none of the simulated individuals have cancer at the start of the model. This ensures that the model ‘warmed up’ to reflect the number of undiagnosed cancers in the population when screening starts.

Women may stay in the same health state, progress to another or die from breast cancer or other causes. Regression is only allowed from ductal carcinoma in situ (DCIS, stage zero) to no diagnosed breast cancer. Women in any model state could die from other causes, while death from breast cancer could occur only in the clinical phase. Women diagnosed with breast cancer and who survived 6 years after initial diagnosis are considered survivors, and their mortality rate was based on the general female population’s age-specific background mortality. Since the model’s aim is screening evaluation, recurrence was not simulated. The model was developed in Microsoft Excel.

The introduction of mammography screening strategies is expected to affect the preclinical phase of disease progression. Once cancer is detected clinically by symptoms or pre-clinically by screening, treatment is expected to be given according to institutional treatment guidelines.

### Analytics

#### Base-case analysis

Over a lifelong time horizon, the model predicts the long-term expected life years (LYs), expected quality-adjusted life years (QALYs) and expected total lifetime costs, as well as the incremental cost-effectiveness ratio (ICER) expressed in US$ per LY gained and incremental cost-utility ratio (ICUR), expressed in US$ per QALY gained. The model also predicted breast cancer incidence (total number of cases and per age group) and mortality (number of deaths) with current and recommended strategies. A willingness-to-pay threshold of US$42 000–US$56 000 per QALY was used as a decision rule.^40^ This threshold was used because there is no national incremental cost-effectiveness threshold for Jordan.

The base-case results of the proposed strategies, compared with the current situation (reference strategy), were presented as an efficiency (cost-effectiveness) frontier to show the associated costs and benefits of all strategies and to illustrate the most cost-effective strategies for Jordan. Some strategies will be dominated because they offer less benefit and entail higher costs than comparator strategies. On the other hand, strategies with higher ICERs than the next most effective alternative are called extended-dominated strategies. Both dominated and extendedly dominated strategies will be eliminated.^41^ The efficiency frontier is the line connecting non-dominated points on a cost-effectiveness plane. Points above the line are considered not cost-effective strategies.^41 42^

#### Model validation

We conducted face validity to ensure that the model represents the natural history of the disease. We conducted internal validation through model verification (including debugging and plausibility checks). We assessed external validity by comparing the model’s results to actual data using external targets other than the calibration targets.^43^ In addition, cross-validation was conducted by reviewing published modelling studies that addressed the same research problem.^44^

### Characterising uncertainty

#### Sensitivity analysis

Both one-way deterministic sensitivity analysis (DSA) and probabilistic sensitivity analysis (PSA) were conducted to assess the model’s robustness and estimate its uncertainty. Utilities, screening costs, discount rates and specificity and sensitivity parameters were tested using DSA. Input variations were based on reported CIs for inputs extracted from the literature, while costs were varied by increasing and decreasing the cost input by 25%. A PSA was conducted to assess the uncertainty in joint parameters; distributions were defined for each input parameter (beta for utilities, sensitivity and specificity; gamma for cost and uniform for transition probabilities). The PSA results were presented as a cost-effectiveness plan and cost-effectiveness acceptability curve (CEAC).

### Patient and public involvement statement

The director of the JBCP is one of the co-authors and a key stakeholder in this project. The input involved describing the current breast cancer screening situation, providing data on screening attendance and participating in suggestions for future organised screening strategies, including an assumed attendance rate of 50%.

## Results

### Study parameters

Online supplemental tables 1–3 show the input parameters for the model. The tables show the mean input values along with ranges and distributions required for sensitivity analysis.

### Calibration results and model validation

Our model is internally valid, as the predictions matched the epidemiological data used in the model (eg, incidence by age: online supplemental figure 1). In terms of external validation, the model could predict specific external data targets not used in the calibration process (eg, distribution by stage and median age at diagnosis: online supplemental table 4). Regarding cross-validation, the model results were comparable to those for screening strategies in LMICs.^45^ Our model predicted the incidence of breast cancer per age under the current opportunistic screening strategy, and the results were comparable to observed data from the national cancer registry.^46^

### Model’s outcomes: effectiveness results

Around 85–153 additional breast cancer cases are expected to be diagnosed (screen-detected plus clinically detected) with proposed screening strategies compared with opportunistic screening. On the other hand, the number of deaths is expected to decrease, reaching its lowest level with screening strategy 6. Screening is expected to impact the distribution of breast cancer stages. Stage 3 and 4 cases are expected to decrease by 3.00%–6.50% for stage 3 and by 3.20%–6.40% for stage 4 compared with the current situation. On the other hand, early-stage cases are expected to increase by 2.40%–6.70% for DCIS and 5.80%–12.10% for stage 1. Online supplemental table 5 shows predicted cases and deaths from each screening strategy.

On average, the current screening strategy (age 40–70, annual, attendance rate 3.5%) was associated with 22.062 discounted LYs and 16.980 discounted QALYs over a lifetime per woman. Regarding the tested screening strategies, our results showed that all five assessed strategies yielded more LYs and QALYs than the current strategy. The highest discounted gains in life expectancy and quality-adjusted life expectancy were observed in screening strategy 6 (40–49 annual; 50–70 biennial; 50% attendance rate), followed by strategy 4 (40–70, biennial, 50% attendance rate). Online supplemental table 6 shows the discounted and undiscounted results of LYs and QALYs.

### Model’s outcomes: cost-effectiveness results

Online supplemental table 6 shows the base case cost-effectiveness results for the proposed screening strategies compared with the current situation (opportunistic screening). Online supplemental table 7 shows a comparison of each strategy with the second-best alternative. Our results showed that strategy 5 (50–70, biennial, 50% attendance rate) is dominated by strategy 2 (50–65, annual, 50% attendance rate). On eliminating strategy 5 and recalculating ICURs, strategy 1 (40–60, biennial, 50% attendance rate) became extendedly dominated. After several recalculations and eliminating extendedly dominated scenarios, only strategy 3 (50–65, biennial, 50% attendance rate), strategy 4 (40–70, biennial, 50% attendance rate) and strategy 6 (40–49 annual and 50–70 biennial, 50% attendance rate) remained with an ICUR of US$6734 per QALY, US$18 024 per QALY and US$61 418 per QALY, respectively. Online supplemental table 8 shows the iterative recalculations of ICURs after removing dominated and extended dominance strategies.

### Sensitivity analysis

#### Deterministic sensitivity analysis

Our results are sensitive to increases or decreases in the discount rate and in the cost of the organised screening package. On the other hand, the results were robust to changes in mammography sensitivity. After changing the attendance rate for all tested strategies to 10%, strategy 3 remained the most cost-effective. Assuming the full potential of the organised screening programme with a 100% attendance rate, strategy 3 (50–65, biennial) remained the most cost-effective. Online supplemental table 9 shows the DSA results, and online supplemental table 10 shows the impact of varying the attendance rate between 10% and 100% on the ICUR.

#### Probabilistic sensitivity analysis

The cost-effectiveness plane for the six assessed strategies, along with the comparator (the current opportunistic screening strategy), is presented in online supplemental figure 2. It shows an overlapping cloud of ICURs with condensed points rather than scattered, indicating a high level of certainty in the results. Online supplemental figure 3 shows the CEAC. The curves plateau, indicating that incremental benefits might not differ dramatically between strategies. In addition, it reflects that there are some dominant and extended dominance strategies. Therefore, a cost-effectiveness frontier and a cost-effectiveness acceptability frontier (CEAF) might be more suitable to represent the results than a CEAC (online supplemental figures 4 and 5). Online supplemental table 11 presents the probabilistic ICERs for each strategy relative to the next-best alternative, compared with the deterministic ICERs. The probabilistic ICUR for strategy 3 was comparable to the deterministic results. The elimination of dominated and extended-dominance strategies leaves only strategies 3 and 4.

## Discussion

Our study generated evidence of the cost-effectiveness of breast cancer screening within the Jordanian context. All tested strategies were associated with increased benefits compared with the current opportunistic screening strategy. However, all tested strategies assumed a 50% attendance rate, which requires an assessment of the readiness of the Jordanian healthcare system to implement screening strategies to ensure this attendance rate. Strategy 3 (50–65 years, biennial, 50% attendance rate) was the most cost-effective strategy with an ICUR of US$6734 per QALY.

Our results showed that the screening benefit is higher with lower start ages, but not necessarily the cost. This might be explained within the epidemiological context of breast cancer in Jordan. The median age of patients with breast cancer at diagnosis in Jordan is 10 years younger (51 years) than the median age in Western countries (61 years).^4^

Applying a cost-effectiveness threshold of US$42 000–56 000 per QALY, strategy 3 (50–65 years, biennial, 50% attendance rate) and strategy 4 (40–70 years, biennial, 50% attendance rate) are likely to be cost-effective. This threshold applied a precedence-based methodological approach and was based on a study that assessed the cost-effectiveness of an antineoplastic medication rather than screening programmes.^40^ Applying the 2026 GDP per capita for Jordan based on the cost-effectiveness threshold method developed by the WHO generates a threshold of US$16044 per QALY. Thus, only screening strategy 3 (50–65 years, biennial, 50% attendance rate) will be cost-effective under the WHO threshold. However, the WHO cost-effectiveness threshold was criticised because it is not directly related to healthcare budgets or population preferences.^47^ In addition, there is advice against using the GDP-based cost-effectiveness threshold as the only decision rule.^48^ The challenges associated with cost-effectiveness thresholds make the case for applying a multicriteria decision-making approach within our Jordanian context when it comes to public health programmes like breast cancer screening. Decision makers need to consider the cost-effectiveness results of this study in light of the available budget, healthcare system capacity and population preferences.

Our results are consistent with Icanervilia *et al*’s (2022) systematic review of the economic evaluation of mammogram screening in LMICs. The review concluded that mammography screening is considered a cost-effective strategy in LMICs.^45^ Mandrik *et al* (2019) studied the determinants of the cost-effectiveness of breast cancer screening and found that in Western European and North American countries, biannual mammography screening was cost-effective in the age range of 50–69 years, including single-reading using computer-aided detection.^49^ Our study results were comparable with those identified determinants regarding screening age interval and frequency.

The WHO recommends screening strategies according to the country’s healthcare system and available resources. It recommends strategy 4 for well-resourced countries and strategy 5 for limited-resource countries. Our results showed that biennial screening of 40–70-year-old women is an option for Jordan, but it is less cost-effective than strategy 3 (50–65, biennial, 50% attendance rate). In the US, the Cancer Intervention and Surveillance Modelling Network estimates showed that starting mammogram screening at the age of 40 was associated with the greatest benefit. However, the study did not explore the cost-effectiveness of the tested screening strategies.^50^ According to the JBCP, the national breast cancer screening guidelines in Jordan were recently updated to recommend biennial mammography screening starting at age 40. However, this is still under an opportunistic screening context.

Targeting very high attendance rates might not translate to a cost-effectiveness strategy.^51 52^ Therefore, selecting the targeted attendance rate requires balancing the estimated benefit and the long-term cost within the context of available healthcare systems infrastructure.

The Jordanian healthcare system must work systematically to increase the screening attendance rate.^18^ Gradual roll-out of organised screening could be implemented in phases, targeting a 10% attendance rate and reaching a maximum of 50%.

Many studies have tackled the issue of low screening attendance rates in Jordan.^53–55^ There is a need to increase mammography screening utilisation by addressing geographical and social disparities in screening uptake in Jordan.^53^ The organised screening strategies tested in our study assumed a 50% attendance rate; this assumption was not arbitrary. Rather, it was informed by the JBCP’s implementation experience through mobile mammography services, community outreach and facilitated referrals to hospitals. In these settings, when important access barriers such as cost and service navigation are actively reduced, response and screening completion can reach approximately 60–65% among women who are contacted and referred for free mammography services. We therefore considered 50% to be a conservative but plausible attendance level for an organised screening programme in Jordan. This estimate also recognises that, even with free or affordable services, participation is shaped by persistent social and cultural barriers, fear and competing family responsibilities. Accordingly, the modelled increase in attendance reflects the added value of an organised screening strategy over opportunistic screening, rather than the effect of service provision alone. Our results showed that improving attendance needs to be done gradually to balance the required benefit, cost and the healthcare system’s capacity to screen and treat screen-detected cases.

Keeping the opportunistic screening strategy while working on increasing the attendance rate, coupled with regular monitoring of the change of attendance rate during the upcoming 3 years, might also be an option. It might provide a better understanding of how to implement organised screening. For example, in South Korea, opportunistic and organised screening are both available, with varying uptake preferences by the target population associated with their socioeconomic status and lifestyle.^56^ Jordan might apply a similar hybrid model through which organised screening is launched with a planned gradual increase in target attendance rate. Opportunistic screening remains an option for the population segments that prefer out-of-pocket private service providers. Future research in Jordan can test the impact of interventions that can be used to improve attendance rate and identify the most cost-effective strategy.

Applying an organised screening strategy in Jordan’s fragmented healthcare sector is challenging, particularly in setting mammography screening costs and coordinating screening. There is a need to set up a breast cancer mammography screening package that can be implemented across a network of institutions from different healthcare sectors: public, private and not-for-profit.

A major strength of our study is developing a cost-effectiveness model based on the national context and available data. The model engine can be updated and adapted using new data. However, there were limitations to the available processed national data that can be used in the model. Nevertheless, our experience with building a decision-analytical model in our study highlighted the need for more observational studies tackling breast cancer screening programme outcomes. Moreover, we used national EQ-5D-3L data to calculate the utilities of the general population. The EQ-5D-3L data were taken from the unpublished results of the Jordanian EQ-5D-3L valuation study.^32^ However, utilities per cancer stage were taken from the literature. It is recommended to generate EQ-5D-3L utilities per breast cancer stage based on the Jordanian value set and assess the impact of using those utilities on the results.

Our study encountered several limitations: we used some data from the cancer registry of KHCC, a disease-specific institution where more state-of-the-art health technologies are available as compared with other national treatment facilities in secondary-care institutions. This might have diminished the impact of screening on mortality. Moreover, we applied a state transition cohort model that does not account for differences in patients’ characteristics. In addition, we faced limitations in finding some of the required local data to inform the model’s input parameters.

### Future recommendations

Assessing the impact of attendance rate on conducting mammography screening might be an unmet research need. In addition, testing the impact of invitations to screening on attendance rates might provide essential information about both the capacity to expand screening programmes and the deeper reasons for non-attendance. Moreover, it is worth assessing the cost-effectiveness of risk-based screening that targets a specific population rather than the average-risk woman. Also, there is a need to conduct a 5-year budget impact analysis of the recommended screening strategies to accompany and complement this economic evaluation study. Health economists at the Centre for Drug Policy and Technology Assessment at KHCC developed a Shiny application for budget impact analysis that decision-makers can use to assess the financial implications of implementing organised cancer screening programmes in countries with limited resources, using breast cancer as an example.^57^

## Conclusion

Organised screening is associated with gains in LYs and QALYs across different scenarios compared with opportunistic screening. Higher attendance rates lead to greater benefits but are not necessarily cost-effective, all other things being equal, under a cost-effectiveness threshold of US$42 000–56 000 per QALY. Therefore, our study results suggest that screening strategy 3 (50–65 years, biennial, 50% attendance rate) and screening strategy 4 (40–70, biennial, 50% attendance rate) are likely to be cost-effective relative to opportunistic screening with ICURs below US$42 000 per QALY in the Jordanian context.

## Supplementary material

10.1136/bmjopen-2025-108486 online supplemental file 1

10.1136/bmjopen-2025-108486 online supplemental file 2

## Data Availability

Data are available upon reasonable request.
