# Supplementary material for: Cost-effectiveness of organised versus opportunistic mammography breast cancer screening in low-resourced country
Source: BMJ Open. 2026 Aug 17;16(8):e108486. doi: 10.1136/bmjopen-2025-108486 (PMC13504863; doi:10.1136/bmjopen-2025-108486)
Supplement: Supplementary file 1 [file bmjopen-2025-108486.reviewer_comments.pdf]

PEER REVIEW HISTORY

BMJ Open publishes all reviews undertaken for accepted manuscripts. Reviewers are asked to complete a checklist review form and are provided with free text boxes to elaborate on their assessment. These free text comments are reproduced below.

ARTICLE DETAILS

Title (Provisional)

Cost Effectiveness of Organized versus Opportunistic Mammography Breast Cancer Screening in Low-Resourced Country

Authors

Al Rabayah, Abeer; Mandrik, Olena; Mansour , Asem; Al-Ajlouni, Reem; Haddad, Tala; Abdel-Razeq, Hikmat; Shamieh, Omar; Chalkidou, Kalipso; Sullivan, Richard; Gheorghe, Adrian

---

VERSION 1 - REVIEW

**Reviewer**	**1**
**Name**	**Dack, Kyle**
**Affiliation**	**University of Bristol, MRC Integrative Epidemiology Unit**
**Date**	**13-Oct-2025**
**COI**	**None**

Reporting adherence: The manuscript currently does not include a checklist to demonstrate adherence to best reporting practices. As the focus is primarily on economic evaluation, I suggest CHEER is suitable. There are some statistical and methodological details required by CHEER which are currently absent, so it would strengthen the transparency of the analysis apply this reporting guideline. I suggest also considering to reorganize the method subheadings to mimic the CHEER items, which may make it easier to locate information.

Abstract:

1. The abstract methodology would benefit from stating the setting, and leading the methods with an explanation of the level of data (i.e. aggregated) and the data sources. The current reading may make readers think that individual-level data was used at some point.

2. The results convey the general finding of organised screening being more cost effective, but there are ambiguities due to the phrasing/grammar which limit the clarity:

-Line 171. This result requires a comparator and the discounting context to be interpretable.

-Line 173: I think the sentence is missing an ending.

-“Screening at age 50-65 years, once every two years, 50% attendance rate demonstrated the highest value.” I suggest adding statistical evidence from the models to support this claim. Possibly it is the next sentence, but there is a full stop in between, so I am unsure.

-Line 175-6. Similar to the above I am not sure which comparison this relates to, and suggest linking up these sentences more.

-Line 179. The final sentence is quite vague, referring to “some screening strategies”, and it would be better if specific examples were highlighted.

Introduction:

3. The introduction is clear, including a strong rationale, and a major strength is that it focuses on the context in Jordan. I do not have suggestions.

Methods:

4. Patient and public involvement: I suggest elaborating in this section, e.g. how was the director a contributor to this project – what specific aspects were they involved with.

5. Line 276-277. I suggest here and elsewhere moving the dollar sign to before the number, to follow the conventions of how this is currency is usually described, and to avoid misunderstandings (for a moment I thought this was saying $16-18).

6. Line 284. I suggest adding that it used aggregate data, as opposed to individual-level data.

7. Line 290. Information about the data is currently dispersed across many sections. Prior to getting into the analysis, it would help readers to report information on the data sources in a single section, e.g. which sources were used for which data. This should be reported first because it affects how to interpret the later statistical sections.

8. Line 294. The model figure is helpful but some readers may prefer to read a summary in the text itself, which I suggest adding.

9. Line 316. Similar to the above comment, it would be helpful to include in-text a summary of the comparison strategies. The reference strategy is described as opportunistic screening, but what are the practicalities of this – under what circumstances does screening occur?

10. The statistical methodology – as reported – appears sound, and I do not have concerns. However, there are a lot of specifics missing which impair the replicability and transparency of the analysis. As mentioned above – this includes the data source in terms of what was taken from each source, the timeframes, and how this maps to the model. But also the model structure and analytical decision making, for example the assumptions, validations, uncertainty handling. I have not gone into full detail as much is covered in CHEER and the CHEER elaboration document found here: https://pubmed.ncbi.nlm.nih.gov/35017145/

Results

11. Line 411, 413, 414: I suggest supporting these claims with specific metrics demonstrating the accuracy.

12. Line 412: Supplementary table 6 does not contain this information, and no table 6 is present in the main document.

13. Line 420. I have highlighted this line but the following comments apply to much of the subsequent reporting. There are significant deviations from CHEER reporting requirements which impair the interpretability of the results. In particular:

-An uncertainty measure for each point estimate, e.g. 95% uncertainty intervals.

-Incremental results relative to the comparator.

-The time context and time units

-Over precision in the number of DP. Line 420. I suggest rounding these findings to 1 or 2 dp because the 3rd dp will be highly unstable, plus lacks clinical significance.

14. Line 444: There are two different notation formats used for $ USD here, and it deviates from an earlier line where the dollar sign was after the number. I suggest applying a single format throughout e.g. $number USD.

15. Table 2. I suggest inserting references into the final column of the table for those who wish to identify these recommendations.

16. Table 3. Please add notes to clarify the abbreviations to enhance readability. In the first column I suggest removing the "_" underscores.

**Reviewer**	**2**
**Name**	**Auguste, Peter**
**Affiliation**	**University of Warwick Warwick Medical School**
**Date**	**13-Feb-2026**
**COI**	**None**

Authors used an economic model to assess the cost-effectiveness of several organised screening strategies compared to opportunistic screening with mammograms to diagnose breast cancer. The illustrative model structure was reported along with the inputs required for the economic evaluation framework. The analysis was undertaken from the health payer perspective over a lifetime horizon and the results reported in terms of an ICER expressed as cost per LYG and cost per QALY.

In general, the paper adhered to best practice guidance in terms of reporting and methodological quality, but there were suggestions, and queries for clarification, which I have outlined in each section.

Abstract

The abstract is well written and provides sufficient information about the conduct of the economic analysis. However, in the methods section it would have been more useful to state

where test accuracy information was obtained. Perhaps the additional information in brackets could be reduced/summarised.

It may be more common to write US$ rather than $US.

In the results section of the abstract, the authors reported the expected yield in terms of LYs and QALYs, but the expected mean costs are not reported, '… mean discounted cost of.'

The authors concluded that some strategies appear cost-effective under a threshold between US$42-US$56,000 per QALY. These results do not appear in the abstract. Also, would it be more appropriate to include the PSA results? What is the probability of these strategies being cost-effective at different willingness-to-pay thresholds?

Introduction

The introduction is well-written and structure and introduces readers to the problem at hand. However, is there more recent data on the prevalence of breast cancer? Additionally, it would be useful to know what proportion of breast cancer cases are identified in the late stages and information about the 5-year survival rate would be useful.

The authors have mentioned that a low screening attendance rate is a challenge. Perhaps it would be useful to know what the barriers to uptake and what interventions have been tried.

Methods

The authors mentioned that the director of the JBCP is a co-author, but stating this isn't sufficient. What was their input to the study design, economic analysis etc.?

The model structure was adapted from previous breast cancer models. Please can the authors provide the references to these previous breast cancer models? Did the authors undertake a systematic review of the literature? What was the rationale for selecting this/these models? It isn't clear what previous models were identified and what adaptations were made.

My reading of Table 1 of the Supplementary material is that the probability of regressing from undetected pre-clinical DCIS to No cancer is higher than the probability of women progressing from no cancer to undetected pre-clinical DCIS. Is this correct?

Test sensitivity and specificity inputs were obtained from the literature; however, in my copy of the supplementary material no references were provided. Are the authors assuming the test sensitivity and specificity of mammography is the same for pre-clinical and clinical phases? Authors stated beta distributions were used for sens/spec in the PSA, but the parameters (alphas and betas) were not reported. Additionally, these ?CIs look narrow.

The authors may want to update reference 29 with the full paper published.

It was assumed that 10% of screened women are expected to receive additional mammography and 25% of screened women are referred for MRI and biopsy. I presume that

the same test accuracy for the second mammography is the same as the first. Additionally, MRI and biopsy are 100% accurate. If so, these assumptions should be clearly stated and if not, the values used should be reported.

Following diagnosis and treatment, women who survived for five years were assumed to follow general population survival. This may be a strong assumption, especially for people with stage IV cancer. It might have been more appropriate to fit parametric curves to data from Abdel-Razeq et al., 2020, then extrapolate for the lifetime horizon and select the best parametric curve for each breast cancer stage. The current approach may overestimate overall survival, especially for women treated for stage IV breast cancer.

Table 2 presents health state utility values. Is it correct that for the 50-54 age group, the utility value (0.696) is lower than the utility values for the older age groups? The authors stated that disutility due to screening was not included in the economic analysis, but what about disutility associated with a false positive result?

The economic analysis included annual follow-up costs for women diagnosed with breast cancer. Are these costs applied up to the 5-years following diagnosis (in line with the assumption that women who survived five years following diagnosis will have general population mortality) or for the duration of the model time horizon?

Analysis

Please can the authors explain their rationale for having a starting age of 20 years (pg 18, line 372) given that opportunistic screening is annually from 40 years and above?

The base-case analysis is based on comparing several strategies against annual opportunistic breast screening, with an attendance rate of 50% and 3.5%, respectively. Is the 50% attendance rate an assumption? If so, what evidence does the authors have to support that with these proposed strategies the attendance rate would increase? Several key input parameters have been included in the sensitivity analysis. However, it would have been useful to explore the impact of varying the proportion of women who are expected to get additional mammography and those referred for MRI or biopsy. It isn't obviously clear about the costs associated with undergoing an MRI or biopsy. Apologies if I have missed it. In my copy of the manuscript and additional information reported, there is no cost-effectiveness acceptability curve or frontier.

Results

It would have been helpful to present results on true positive, true negative, false positive and false negative cases?

Many thanks for including Table 4 with the model's validation results. It would be even more transparent if the other breast cancer stages observed and predicted are reported.

The authors reported the total predicted cancers, but it would have been useful to see disaggregated results with the cancers detected (screen-detected and clinically detected)

and missed by each strategy. Additionally, it would have been useful to see disaggregated results in terms of life year results by stage. Comparing Scenario 6 (50% attendance) with the standard practice (3.5% attendance), there is a difference of just over 60 breast cancer deaths. I am surprised by the incremental difference in terms of life years (discounted 0.03 LYG) between these two strategies.

In Table 4, the authors presented the incremental results based on the cost per QALY. However, I think these need to be re-checked in terms of dominated and extendedly dominated strategies. Based on the absolute values, scenario 5 is either absolutely dominated or extendedly dominated by the comparison between scenario 3 and scenario 1. Additionally, there are other extendedly dominated strategies (scenario 1 and 2) in the table.

The authors may want to consider the following text… Strategy three was the most cost-effective strategy with an ICUR of US$ 6,734 per QALY when compared to the opportunistic screening strategy.

I found Table 6 challenging to follow. Perhaps the authors may want to consider pairwise comparisons and presenting the results in the form of tornado diagrams, but I appreciate that this may take more time. Additionally, I think other parameters could have been included in the one-way sensitivity analysis. For example, varying the attendance rates for the comparator and the strategies. Additionally, the percentage of women who are expected to get additional mammography and the percentage of women who receive MRI/biopsy.

Unfortunately, I am unable to provide comment on the results of the PSA as no CEAC/frontier is included in the submission. It would have been helpful for the authors to report tabulated PSA results similar to Table 4 to see how the results compare to the deterministic results. The authors stated that scenario three is likely to be cost-effective. But what is the probability of it being cost-effective?

Discussion

The discussion is well written and structured. My first comment on this section relate to more weight/discussion should be given to the 50% attendance rate and how this would be achieved.

The authors stated that the implementation action plans need careful assessment of the infrastructure that can absorb the increased number of breast cancer cases. I would have thought that the number of breast cancer cases is fixed within the model, but some strategies there may be a stage shift when cases are detected.

Reviewer **3**

Name **Huxley, Nicola**

Affiliation **Monash University, Centre for Health Economics, Monash Business School**

**Date**	**17-Feb-2026**
**COI**	**None**

The authors present an interesting economic evaluation of screening approaches in breast cancer that potentially adds to the literature.

However, I have some concerns with the justification of the paper and the clarity of the write up.

The abstract could do with being a bit clearer. The setting and perspective were not clear. The results included descriptions that would be better placed in the background/methods, such as describing the current screening strategy were included in the results. The results presented in the abstract were not intuitive (the presented ICER did not appear to follow from the presented results- likely due to the use of extended dominance, which is not clear in the abstract), and the WTP threshold in the conclusion is not described in the methods.

In the introduction and discussion there are often sentences that contradict or do not follow. E.g., Lines 228-229 discuss the context of low HDI, but 230-231 describe Jordan as high HDI (so how does the low HDI context apply?); Lines 499-500 in the discussion are unclear (what does screening being cost-effective in general for LMIC and specifically for UMIC mean?); Lines 516-517 discuss annual screening and apply the conclusion to uptake, and the logic of this needs to be set out more clearly.

As a reader unfamiliar with the HDI and with the Jordan context, a stronger case for a separate economic analysis for Jordan and how healthcare is paid for (and later in the methods which costs are included in your analysis- out of pocket and insurance, or just government healthcare costs) would be appreciated. How does the $42,000 cost of treating mBC compare to the cost of treating non-mBC? $42,000 seems low compared to the cost of treatment in other countries.

The choice of screening strategies is unclear. It appears guidelines recommend annual screening, but there is low adherence to these recommendations. The paper does not adequately explain the choice of the alternative screening (the discussion mentions other WHO guidelines, Lines 507-508, but these are not presented earlier) and why these would be better adhered to. Indeed, based on the current recommendations, it may be more reasonable to explore the cost-effectiveness of adherence strategies (for clinicians and patients) rather than alternate screening strategies with an assumed 50% adherence rate. As the authors discuss, there are also implications for accessibility and scaling up of testing for the wider healthcare sector which need to be explored with screening strategies.

Sensitivity and specificity appeared to be for mammograms only, though costs mentioned weighting for ultrasound. The accuracy of the screening type varies for women, particularly with age. It was also unclear how false positives and false negatives were treated in the model. Please could the authors expand upon this. False positive patients may experience

quality of life decrement and undergo unnecessary procedures, whereas false negative patients will require future testing after presence of symptomatic disease with worse prognosis.

The steps to calibrate the transition probabilities were unclear, perhaps an example could be presented in the appendix to make the paper more reproducible.

Similarly, the utilities could not be verified from the sources. Please clarify how these values were calculated from the published sources, and if unpublished data can not be presented, be clear about this. Furthermore, the approach to utilities meant the improvements in quality of life had the least impact for women aged 50-54 (general population utility was lowest at this age), which coincides with the median age of breast cancer diagnosis in Jordan (51). The authors should consider how the utility by age may have affected the effectiveness of the strategies, and whether this was plausible, e.g., the cross-sectional nature of the utility estimates may not translate to utility over an individual's lifetime.

Please present the distribution of the model parameters used in the PSA in the appendix to make the analysis more reproducible.

It would be good to describe the choice of willingness to pay threshold in the methods and why it was chosen. Lines 484-490 in the discussion were confusing given Scenario 3 was described as meeting both thresholds.

I did want to note that the validation methods were very clearly stated and reasonable, though they might be easier to discuss following the main results or in the discussion when comparing to external sources.

I think extended dominance results can be a little tricky for readers. It might be better to present all ICERs versus current practice and then the extended dominance analysis

Please complete a CHEERS checklist as recommended by BMJOpen and make sure the paper aligns with these guidelines (e.g., state whether a health economic plan was written prior to the analysis)

Overall, I think the paper has the potential to be a good publication and thank the authors for their submission.

VERSION 1 - AUTHOR RESPONSE

Reviewer: 1
Dr. Kyle Dack, University of Bristol
Comments to the Author:

Reporting adherence: The manuscript currently does not include a checklist to demonstrate adherence to best reporting practices. As the focus is primarily on economic evaluation, I suggest CHEER is suitable. There are some statistical and methodological details required by CHEER which are currently absent, so it would strengthen the transparency of the analysis

apply this reporting guideline. I suggest also considering to reorganize the method subheadings to mimic the CHEER items, which may make it easier to locate information.

Response: Thank you for your comment. We attached the CHEERS checklist to the revised submission. In addition, we organized the methods section to match the CHEERs items.

Abstract:
1. The abstract methodology would benefit from stating the setting, and leading the methods with an explanation of the level of data (i.e. aggregated) and the data sources. The current reading may make readers think that individual-level data was used at some point.

Response: Thank you for your comment. The first sentence (line 161) of the methods section indicate that we used a decision-analytic model. Sources of information were mentioned in lines 168-169.
2. The results convey the general finding of organised screening being more cost effective, but there are ambiguities due to the phrasing/grammar which limit the clarity:
-Line 171. This result requires a comparator and the discounting context to be interpretable.
-Line 173: I think the sentence is missing an ending.
-"Screening at age 50-65 years, once every two years, 50% attendance rate demonstrated the highest value." I suggest adding statistical evidence from the models to support this claim. Possibly it is the next sentence, but there is a full stop in between, so I am unsure.
-Line 175-6. Similar to the above I am not sure which comparison this relates to, and suggest linking up these sentences more.
-Line 179. The final sentence is quite vague, referring to "some screening strategies", and it would be better if specific examples were highlighted.

Response: Thank you for your comment. We wrote the results section again, taking into consideration all your comments on this part of the abstract.

Introduction:
3. The introduction is clear, including a strong rationale, and a major strength is that it focuses on the context in Jordan. I do not have suggestions.

Methods:
4. Patient and public involvement: I suggest elaborating in this section, e.g. how was the director a contributor to this project – what specific aspects were they involved with.

Response: Thank you for your comment. The director of the Jordan Breast Cancer Screening Program provided information on screening attendance and the future perspective of breast cancer screening in Jordan, and offered suggestions for possible screening strategies to test. The director also reviewed the manuscript and was involved in each step of the project as a co-author and main stakeholder.

5. Line 276-277. I suggest here and elsewhere moving the dollar sign to before the number, to follow the conventions of how this is currency is usually described, and to avoid misunderstandings (for a moment I thought this was saying $16-18).

Response: Thank you for your comment. The position of the dollar sign is now corrected.

6. Line 284. I suggest adding that it used aggregate data, as opposed to individual-level data.

Response: Thank you for your comment. This is a model-based study that used mainly secondary data and was populated with aggregated, not individual-level, data. Usually, model-based studies use data extracted from different sources. In this study, we used published secondary data and real-world data from cancer registry, claims data, and epidemiological data. We modified the study design to clearly reflect the utilization of a model rather than using individual-level data.

7. Line 290. Information about the data is currently dispersed across many sections. Prior to getting into the analysis, it would help readers to report information on the data sources in a single section, e.g. which sources were used for which data. This should be reported first because it affects how to interpret the later statistical sections.

Response: Thank you for your comment. We reorganized the methods section to align with the CHEERS checklist.

8. Line 294. The model figure is helpful but some readers may prefer to read a summary in the text itself, which I suggest adding.

Response: Thank you for your comment. The summary of how women will move between the health states is written under the decision analytical modelling method section, page 15. After adjusting the methods section to match the CHEERs, the summary is now available under rational and description of the model section, page 19.

9. Line 316. Similar to the above comment, it would be helpful to include in-text a summary of the comparison strategies. The reference strategy is described as opportunistic screening, but what are the practicalities of this – under what circumstances does screening occur?

Response: Thank you for this comment. Page 16 includes a specific section titled "assessed screening strategies." Under this section, the reference strategy, opportunistic screening, is described and linked to national screening guidelines. Also, this section refers to Table 2 in the manuscript, which details each screening strategy. After adjusting the methods section to match the CHEERs, the summary is now available under Interventions and comparator sections, page 16.

10. The statistical methodology – as reported – appears sound, and I do not have concerns. However, there are a lot of specifics missing which impair the replicability and transparency of the analysis. As mentioned above – this includes the data source in terms of what was taken from each source, the timeframes, and how this maps to the model. But also the model structure and analytical decision making, for example the assumptions, validations, uncertainty handling. I have not gone into full detail as much is covered in CHEER and the CHEER elaboration document found here: https://pubmed.ncbi.nlm.nih.gov/35017145/

Response: Thank you for this comment. We adjusted the methods section to fully align with the CHEERS checklist and so correspond to the standard reporting practice for Health Economics studies.

Results

11. Line 411, 413, 414: I suggest supporting these claims with specific metrics demonstrating the accuracy.

Response: Thank you for your comment. Table 4 in the supplementary material shows the alignment between the model predications and the observed data from KHCC and national cancer registries. Also figure, 1 shows the validation of the calibration results (Observed versus modelled).

Table (4). Model's validation results

Validation Target	Observed (national cancer registry data 2018)	Model predictions
Breast cancer deaths	342	352.3
Median age at diagnosis (years)	51	50.5
DCIS	2%	1.4%
Stage 4	13%	12.3%

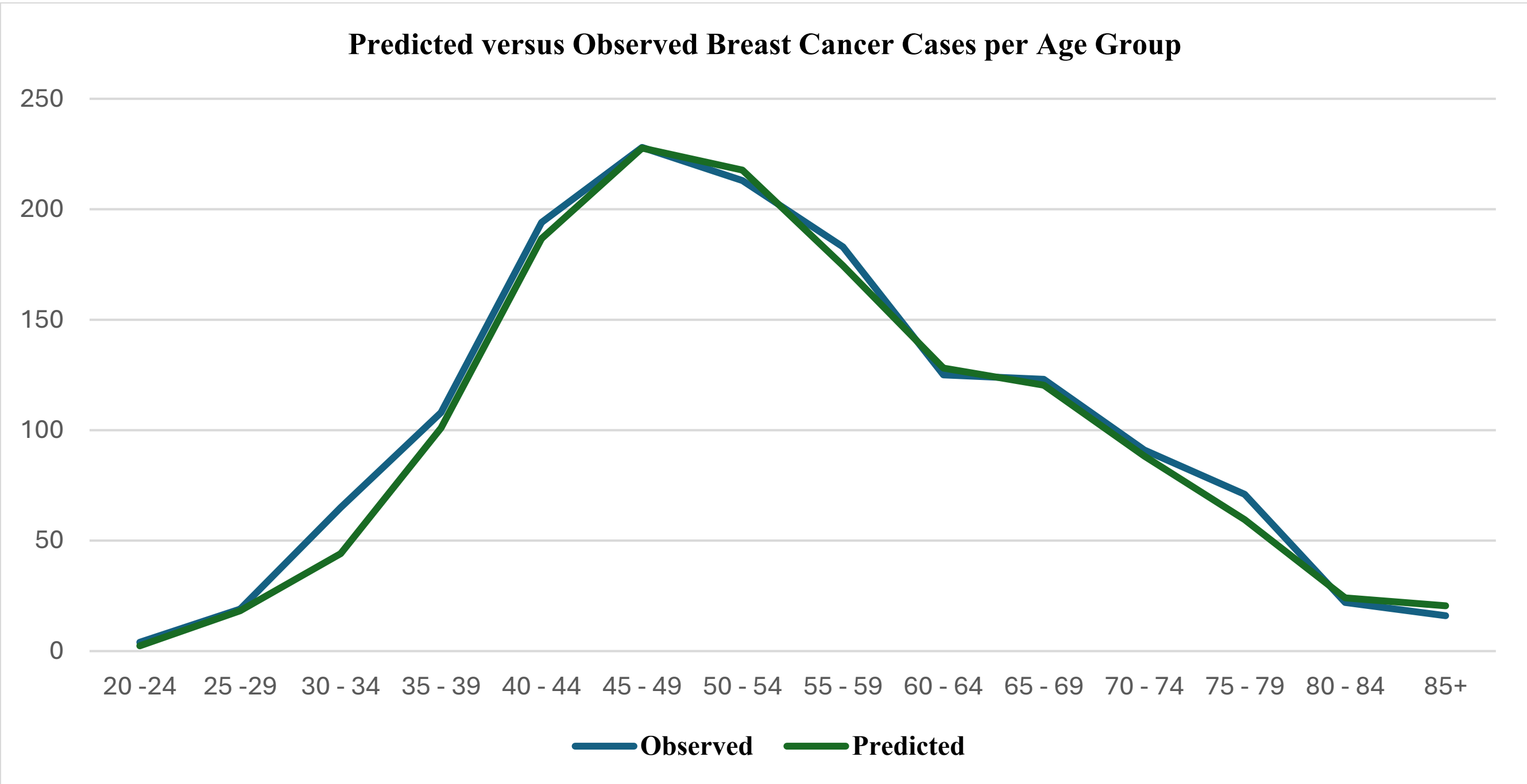

Figure (1). Model calibration results (validation)

12. Line 412: Supplementary table 6 does not contain this information, and no table 6 is present in the main document.

Response: Thank you for your comment. We agree with your comment. It should be table 4 and figure 1, not table 6.

13. Line 420. I have highlighted this line but the following comments apply to much of the subsequent reporting. There are significant deviations from CHEER reporting requirements which impair the interpretability of the results. In particular:
-An uncertainty measure for each point estimate, e.g. 95% uncertainty intervals.
-Incremental results relative to the comparator.
-The time context and time units
-Over precision in the number of DP. Line 420. I suggest rounding these findings to 1 or 2 dp because the 3rd dp will be highly unstable, plus lacks clinical significance.

Response: Thank you for your comment. The results section is divided into clinical effectiveness, cost effectiveness, and sensitivity analysis sections. The incremental results are reported under the cost-effectiveness section. The time horizon is a lifetime, so all results are over that horizon to capture the ultimate costs and benefits. The uncertainty of point estimates will be added to the parameter tables. The 95% uncertainty intervals for some parameters, such as the screening's specificity and sensitivity, are reported. In addition, we rounded the numbers to 2 decimal places.

14. Line 444: There are two different notation formats used for $ USD here, and it deviates from an earlier line where the dollar sign was after the number. I suggest applying a single format throughout e.g. $number USD.

Response: Thank you for this comment. We standardized the currency format in the paper.

15. Table 2. I suggest inserting references into the final column of the table for those who wish to identify these recommendations.

Response: Thank you for your comment. We added the requested references.

16. Table 3. Please add notes to clarify the abbreviations to enhance readability. In the first column I suggest removing the "_" underscores.

Response: Thank you for your comment. We modified Table 3 based on your recommendations.

Reviewer: 2
Mr. Peter Auguste, University of Warwick Warwick Medical School
Comments to the Author:
Authors used an economic model to assess the cost-effectiveness of several organised screening strategies compared to opportunistic screening with mammograms to diagnose breast cancer. The illustrative model structure was reported along with the inputs required for the economic evaluation framework. The analysis was undertaken from the health payer

perspective over a lifetime horizon and the results reported in terms of an ICER expressed as cost per LYG and cost per QALY.

In general, the paper adhered to best practice guidance in terms of reporting and methodological quality, but there were suggestions, and queries for clarification, which I have outlined in each section.

Abstract
The abstract is well written and provides sufficient information about the conduct of the economic analysis. However, in the methods section it would have been more useful to state where test accuracy information was obtained. Perhaps the additional information in brackets could be reduced/summarised.

Response: Thank you for your comment. We added the source of the test accuracy to the abstract.

It may be more common to write US$ rather than $US.
Response: Thank you for your comment. We adjusted and standardized the currency formatting in the paper to become US$.

In the results section of the abstract, the authors reported the expected yield in terms of LYs and QALYs, but the expected mean costs are not reported, '… mean discounted cost of.'

Response: Thank you for this comment. We adjusted the results section to include the mean cost as well.

The authors concluded that some strategies appear cost-effective under a threshold between US$42-US$56,000 per QALY. These results do not appear in the abstract. Also, would it be more appropriate to include the PSA results? What is the probability of these strategies being cost-effective at different willingness-to-pay thresholds?

Response: Thank you for this comment. We adjusted the results section to reflect which strategies are cost-effective. However, the PSA resulted in a plateau, according to the CEAC figure, most probably driven by a dominated and extended-dominance strategy.
Therefore, we reported in the abstract that we reported results as a cost-effectiveness frontier along with the deterministic sensitivity analysis results. In the main text results section, more details are provided, and all related figures are included in the supplementary material.

Introduction
The introduction is well-written and structure and introduces readers to the problem at hand. However, is there more recent data on the prevalence of breast cancer? Additionally, it would be useful to know what proportion of breast cancer cases are identified in the late stages and information about the 5-year survival rate would be useful.

Response: Thank you for your comment. It is mentioned in the introduction section that around 14% of diagnosed breast cancer cases present in stage 4 (metastatic stage). In addition, there is information on the cumulative prevalence and incidence of breast cancer. We modified the introduction by adding the 5-year survival rate.

The authors have mentioned that a low screening attendance rate is a challenge. Perhaps it would be useful to know what the barriers to uptake and what interventions have been tried.

Response: Thank you for your comment. We updated the introduction [lines 259-262] to include data on barriers to screening uptake in Jordan and on current interventions to increase the attendance rate.

Methods

The authors mentioned that the director of the JBCP is a co-author, but stating this isn't sufficient. What was their input to the study design, economic analysis etc.?

Response: Thank you for your comment. The input involved describing the current breast cancer screening situation, providing data on screening attendance, and participating in suggestions for future organized screening strategies, including an assumed attendance rate of 50%. In addtion, the director of the JBCP was among the clinical experts and stakholders who validated the model inputs.

The model structure was adapted from previous breast cancer models. Please can the authors provide the references to these previous breast cancer models? Did the authors undertake a systematic review of the literature? What was the rationale for selecting this/these models? It isn't clear what previous models were identified and what adaptations were made.

Response: Thank you for your comment. It is reference 35 : Schiller-Fruehwirth I, Jahn B, Einzinger P, Zauner G, Urach C, Siebert U. The Long-Term Effectiveness and Cost Effectiveness of Organized versus Opportunistic Screening for Breast Cancer in Austria. Value Health. 2017;20(8):1048-57.

We did not conduct a systematic review from scratch, but we reviewed published systematic reviews on the cost-effectiveness of breast cancer screening, reference 23: Schiller-Frühwirth IC, Jahn B, Arvandi M, Siebert U. Cost-Effectiveness Models in Breast Cancer Screening in the General Population: A Systematic Review. Appl Health Econ Health Policy. 2017;15(3):333-51.

We adapted the model structure from the study by Schiller-Fruehwirth I et al. (reference 35) because it addresses a decision problem similar to ours. It compared an organized strategy to an opportunistic strategy. We adapted only the model's structure and health states, but we applied a different model. We use a cohort state transition model rather than an individual-level (microsimulation) state-transition model. Reference 23, the systematic review by Schiller-Frühwirth et al., showed that state-transition cohort decision-analytic models are the most commonly used type. In addition, it is suitable for our decision problem.

My reading of Table 1 of the Supplementary material is that the probability of regressing from undetected pre-clinical DCIS to No cancer is higher than the probability of women progressing from no cancer to undetected pre-clinical DCIS. Is this correct?

Response: Thank you for your comment. Yes, this is correct.

Test sensitivity and specificity inputs were obtained from the literature; however, in my copy of the supplementary material no references were provided. Are the authors assuming the test sensitivity and specificity of mammography is the same for pre-clinical and clinical phases? The authors stated beta distributions were used for sens/spec in the PSA, but the parameters (alphas and betas) were not reported. Additionally, these ?CIs look narrow.

Response: Thank you for your comment. We added the references for specificity and sensitivity to the table in the supplementary material.

The sensitivity and specificity apply only to the pre-clinical health states (those without symptoms). Those in the clinical stage will be symptomatic, and cancer will be detected by diagnosis, not screening.

We updated all tables in the supplementary file to include all PSA parameters as recommended by the CHERRS checklist. The sensitivity and specificity parameters and their 95% confidence intervals are taken from the literature.

The authors may want to update reference 29 with the full paper published.

Response: Thank you for your comment. We updated reference 29, now become reference 31 in the manuscript and reference 5 in the supplementary.

It was assumed that 10% of screened women are expected to receive additional mammography and 25% of screened women are referred for MRI and biopsy. I presume that the same test accuracy for the second mammography is the same as the first. Additionally, MRI and biopsy are 100% accurate. If so, these assumptions should be clearly stated and if not, the values used should be reported.

Response: Thank you for your comment. Yes, we assumed that the test accuracy is the same. Yes, we assumed that the MRI and biopsy are 100% accurate. We updated the methods section with this assumption. Page 17, under the effectiveness section.

Following diagnosis and treatment, women who survived for five years were assumed to follow general population survival. This may be a strong assumption, especially for people with stage IV cancer. It might have been more appropriate to fit parametric curves to data from Abdel-Razeq et al., 2020, then extrapolate for the lifetime horizon and select the best parametric curve for each breast cancer stage. The current approach may overestimate overall survival, especially for women treated for stage IV breast cancer.

Thank you for your comment. We fully agree that, from an analytical perspective, our assumption is strong and conducting parametric survival analysis would lead to more precise results; from a decision-making perspective, however, this assumption is conservative for the cost-effectiveness of screening because general population survival overestimates survival the most in the most advanced cancer stages, which screening brings down, so more precision here would make screening appear even more cost-effective than in our base case results. Overall, we would not expect our conclusions to change.

Table 2 presents health state utility values. Is it correct that for the 50-54 age group, the utility value (0.696) is lower than the utility values for the older age groups? The authors

stated that disutility due to screening was not included in the economic analysis, but what about disutility associated with a false positive result?

Response: Thank you for your comment. Those utilities are the EQ-VAS scores collected during the implementation of the EQ-5D valuation study in Jordan. The false positive women will be treated for cancer, and the utility per stage was applied.

The economic analysis included annual follow-up costs for women diagnosed with breast cancer. Are these costs applied up to the 5-years following diagnosis (in line with the assumption that women who survived five years following diagnosis will have general population mortality) or for the duration of the model time horizon?

Response: Thank you for your comment. Yes, these costs were applied up to 5-years following diagnosis.

Analysis
Please can the authors explain their rationale for having a starting age of 20 years (pg 18, line 372) given that opportunistic screening is annually from 40 years and above?

Response: Thank you for your comment. The age of the simulated population at the start is 20 years, because the model simulates the natural history of cancer, where none of the simulated individuals have cancer onset at the model start. This ensures that the model "warmed up" to reflect the number of undiagnosed cancers in the population at the screening start. This approach is common in cancer screening models with natural history disease. The opportunistic screening starts from the age of 40 years.

The base-case analysis is based on comparing several strategies against annual opportunistic breast screening, with an attendance rate of 50% and 3.5%, respectively. Is the 50% attendance rate an assumption? If so, what evidence does the authors have to support that with these proposed strategies the attendance rate would increase? Several key input parameters have been included in the sensitivity analysis. However, it would have been useful to explore the impact of varying the proportion of women who are expected to get additional mammography and those referred for MRI or biopsy. It isn't obviously clear about the costs associated with undergoing an MRI or biopsy. Apologies if I have missed it. In my copy of the manuscript and additional information reported, there is no cost-effectiveness acceptability curve or frontier.

Response: Thank you for your comment. Yes, the 50% attendance rate was a model assumption in the base-case analysis. However, it was not arbitrary. It was based on JBCP's long-term implementation experience, which showed that when screening is actively organized and barriers are reduced, participation increases substantially beyond current opportunistic levels. In particular, JBCP has observed that when women are reached through community outreach and referred for free mammography, approximately 60–65% respond and complete screening. We used this experience as a realistic uptake factor in estimating screening participation in Jordan.
The rationale for expecting attendance to increase under the proposed strategies is that these strategies represent a shift from opportunistic screening to a more structured approach. Opportunistic screening largely depends on individual initiative, healthcare providers'

referrals, and fragmented access, whereas organized screening can improve uptake through invitation and recall mechanisms, community outreach, facilitated referrals, and free or affordable screening. Current national uptake remains low. Therefore, the model assumption of 50% reflects the expected gain from a structured screening program, while remaining conservative relative to JBCP's observed outreach response rates. We selected 50%, rather than 60–65%, because scaling from targeted outreach to a national program would still be constrained by persistent social and cultural barriers, meaning that 50% is better interpreted as an ambitious but plausible upper-range attendance estimate for Jordan. This assumption was the most defensible assumption for the base case in the view of local cancer experts, given that no organized cancer screening is in place in Jordan

Regarding the additional mammogram, MRI, or biopsy, the costs are included in the screening bundle and were not texted separately. Regarding the CEAC, we added it, along with the cost-effectiveness frontier, to the supplementary file. Figures 3 and 4

Results
It would have been helpful to present results on true positive, true negative, false positive and false negative cases?

Response: Thank you for your comment. In the model design, we did not include a separate health state for testing results. Those were taken into consideration within the embedded equations. However, in the future, we can make such a modification, as this model is expected to be used by decision-makers to test any future proposed screening strategies.

Many thanks for including Table 4 with the model's validation results. It would be even more transparent if the other breast cancer stages observed and predicted are reported.

Response: Thank you for your comment. We updated the table to include the remaining breast cancer stages. Stage one was not used as a target, so it is not included in the table.

The authors reported the total predicted cancers, but it would have been useful to see disaggregated results with the cancers detected (screen-detected and clinically detected) and missed by each strategy. Additionally, it would have been useful to see disaggregated results in terms of life year results by stage. Comparing Scenario 6 (50% attendance) with the standard practice (3.5% attendance), there is a difference of just over 60 breast cancer deaths. I am surprised by the incremental difference in terms of life years (discounted 0.03 LYG) between these two strategies.

Response: Thank you for your comment. We updated Table 5 to include disaggregated results of clinically and screen-detected cases. The screening strategies are influencing the cancer stages and are contributing to down-staging, so more patients are diagnosed at an earlier stage with lower mortality risk compared with more advanced cancer stages.

In Table 4, the authors presented the incremental results based on the cost per QALY. However, I think these need to be re-checked in terms of dominated and extendedly dominated strategies. Based on the absolute values, scenario 5 is either absolutely dominated or extendedly dominated by the comparison between scenario 3 and scenario 1. Additionally,

there are other extendedly dominated strategies (scenario 1 and 2) in the table.

Response: Thank you for your comment. We agree with this comment, and we updated the tables to include Table 3. Base case results based on comparing each strategy with the current comparator, Table 4. Base case results, comparing each intervention with the following effective strategy, and Table 5. Recalculation of ICERs after elimination of extended dominance strategies.

The authors may want to consider the following text… Strategy three was the most cost-effective strategy with an ICUR of US$ 6,734 per QALY when compared to the opportunistic screening strategy.
Response: Thank you for your comment.

I found Table 6 challenging to follow. Perhaps the authors may want to consider pairwise comparisons and presenting the results in the form of tornado diagrams, but I appreciate that this may take more time. Additionally, I think other parameters could have been included in the one-way sensitivity analysis. For example, varying the attendance rates for the comparator and the strategies. Additionally, the percentage of women who are expected to get additional mammography and the percentage of women who receive MRI/biopsy.

Response: Thank you for your comment. We updated the sensitivity analysis to include variations in the attendance rate. However, we added a separate table showing the sensitivity analysis results for attendance rates ranging from 10% to 100%. Regarding the percentage of women who are expected to get additional mammography and the percentage of women who receive MRI/biopsy, those percentages were reflected in the cost only and were varied as part of the cost of screening in the sensitivity analysis.

Unfortunately, I am unable to provide comment on the results of the PSA as no CEAC/frontier is included in the submission. It would have been helpful for the authors to report tabulated PSA results similar to Table 4 to see how the results compare to the deterministic results. The authors stated that scenario three is likely to be cost-effective. But what is the probability of it being cost-effective?

Response: Thank you for your comment. We added the CEAC and the frontier to the supplementary file (figures 3 and 4). In addition, we added the new Table 6 to the main manuscript file that compares deterministic and probabilistic results for each strategy.

Discussion
The discussion is well written and structured. My first comment on this section relate to more weight/discussion should be given to the 50% attendance rate and how this would be achieved.

Response: Thank you for your comment. We agree with your comment. We modified the Discussion to better justify the 50% attendance assumption and to explain the implementation conditions under which such attendance could be achieved.

The 50% attendance rate was selected as a realistic but conservative participation target for a structured screening program in Jordan. It was informed by the Jordan Breast Cancer Program's

field experience through mobile mammography services, community outreach, and facilitated referrals to hospitals. In these settings, when major barriers are actively reduced, particularly cost and access barriers, response and screening completion can reach approximately 60–65% among women who are contacted and referred for free mammography.

At the same time, we considered 50% a more appropriate assumption for a nationally organized screening strategy because not all barriers are financial or geographic. In Jordan, participation in screening is also shaped by social and cultural factors, fear, low perceived risk, and competing family responsibilities. Therefore, even with free or affordable screening, active invitations, navigation, and structured referrals, full uptake should not be expected. For this reason, 50% was intended to reflect the highest plausible attendance under an organized program, while remaining more conservative than the response rates observed in targeted JBCP-supported outreach settings. We revised the Discussion to make this rationale explicit and to note that the recent shift in Jordanian guidance from annual to biennial screening may further improve feasibility and acceptability.

The authors stated that the implementation action plans need careful assessment of the infrastructure that can absorb the increased number of breast cancer cases. I would have thought that the number of breast cancer cases is fixed within the model, but some strategies there may be a stage shift when cases are detected.

Response: Thank you for your comment. Yes, there will be stage shifts, but there will also be more detected cases as the attendance rate increases. More cases in the pre-clinical health state will be detected.

Reviewer: 3
Dr. Nicola Huxley, Monash University
Comments to the Author:
The authors present an interesting economic evaluation of screening approaches in breast cancer that potentially adds to the literature.
However, I have some concerns with the justification of the paper and the clarity of the write up.

Abstract

The abstract could do with being a bit clearer. The setting and perspective were not clear. The results included descriptions that would be better placed in the background/methods, such as describing the current screening strategy were included in the results. The results presented in the abstract were not intuitive (the presented ICER did not appear to follow from the presented results- likely due to the use of extended dominance, which is not clear in the abstract), and the WTP threshold in the conclusion is not described in the methods.

Response: Thank you for your comment. We updated the abstract to reflect the mentioned comments by the reviewer.

Introduction and discussion

In the introduction and discussion there are often sentences that contradict or do not follow.

E.g., Lines 228-229 discuss the context of low HDI, but 230-231 describe Jordan as high HDI (so how does the low HDI context apply?); Lines 499-500 in the discussion are unclear (what does screening being cost-effective in general for LMIC and specifically for UMIC mean?); Lines 516-517 discuss annual screening and apply the conclusion to uptake, and the logic of this needs to be set out more clearly.

Response: Thank you for your comment. We resolved the contradictions and clarified the sentences in the mentioned lines. We removed lines 516-517 because they are not consistent with the idea in this paragraph.

As a reader unfamiliar with the HDI and with the Jordan context, a stronger case for a separate economic analysis for Jordan and how healthcare is paid for (and later in the methods which costs are included in your analysis- out of pocket and insurance, or just government healthcare costs) would be appreciated. How does the $42,000 cost of treating mBC compare to the cost of treating non-mBC? $42,000 seems low compared to the cost of treatment in other countries.

Response: Thank you for your comment. The US$ 42,000 per case; this is an estimate, whereas in some institutions the cost is much higher due to the introduction of new, innovative medications. This number excludes emergency admissions and targeted biological therapies, so yes, it is an underestimate. In some studies, the estimated cost per less severe stage was estimated. In the supplementary material, the mean cost per stage from the three major cancer-treating institutions in Jordan is reported. Thoses numbers was used as inputs in our model. We removed the HDI classification. However, the HDI reflects countries' achievements in key health dimensions (life expectancy, mean years of schooling, and standard of living). Jordan's score indicates it is performing well on this index, but it still faces economic challenges.

In Jordan, we need a separate cost-effectiveness study of breast cancer screening strategies in to determine which strategy provides the greatest benefit for our population, based on our data, and to identify the best use of our limited resources and where to invest in screening. Therefore, national future cancer screening guidelines and any national screening policies will benefit from this study in decision-making, and it will inform future planning and budgeting. In addition, in the introduction, we highlighted the problem of identifying breast cancer cases in late stages, and this is associated with a high economic burden. Therefore, organized screening will contribute to downstaging, which is associated with a better prognosis and lower treatment costs.

The choice of screening strategies is unclear. It appears guidelines recommend annual screening, but there is low adherence to these recommendations. The paper does not adequately explain the choice of the alternative screening (the discussion mentions other WHO guidelines, Lines 507-508, but these are not presented earlier) and why these would be better adhered to. Indeed, based on the current recommendations, it may be more reasonable to explore the cost-effectiveness of adherence strategies (for clinicians and patients) rather than alternate screening strategies with an assumed 50% adherence rate. As the authors discuss, there are also implications for accessibility and scaling up of testing for the wider healthcare sector which need to be explored with screening strategies.

Response: Thank you for your comment.

The choice of screening strategies was based on national recommendations by stakeholders and based on WHO recommendations for LMICs. The interventions sub-section under the methods section clarifies why those strategies were selected.
Yes, we agree that exploring adherence strategies might be very important, as the main challenge for breast cancer screening in Jordan is attendance rate. Furthermore, regarding adherence to new proposed strategies, there is still uncertainty regarding how applying any of these new strategies will influence attendance rate. However, the idea is that having an organized program means there will be more coordination and national communication with eligible women to remind them about the screening. In the future, we can assess the real impact on the attendance rate.
However, exploring several strategies is also useful, as it highlights the impact of the attendance rate and the need to assess whether Jordan's infrastructure can absorb additional cases. It is important for decision makers to use both cost-effectiveness and budget impact to inform their decisions. Therefore, the first author with another colleague from KHCC built a budget impact shinney application for this purpose. We updated the discussion to reflect on this new application. In addition, we will reflect on the importance of assessing adherence strategies in future research.

Sensitivity and specificity appeared to be for mammograms only, though costs mentioned weighting for ultrasound. The accuracy of the screening type varies for women, particularly with age. It was also unclear how false positives and false negatives were treated in the model. Please could the authors expand upon this. False positive patients may experience quality of life decrement and undergo unnecessary procedures, whereas false negative patients will require future testing after presence of symptomatic disease with worse prognosis.

Response: Thank you for your comment. We used sensitivity and specificity that vary with age.
Our model structure was based on simulating the natural history of progression of breast cancer, and then we tested the impact of screening, especially on down-staging. We did not model explicit health states for false positives and false negatives. However, their implications were taken into consideration in the model. The false-positive impact on cost was accounted for by adding the recall cost to the screening cost bundle. We did not account for disutility because we assumed that screening has no impact on HRQoL, this assumption was based on the following evidence: Li L, Severens JLH, Mandrik O. Disutility associated with cancer screening programs: A systematic review. PLoS One. 2019;14(7):e0220148.

Regarding false negatives, its impact was taken into consideration in the model flow, as those cases stayed in the pre-clinical health state because they were missed, as we do not have 100% sensitive mammogram.

The steps to calibrate the transition probabilities were unclear, perhaps an example could be presented in the appendix to make the paper more reproducible.

Response: Thank you for your comment. In the methods section, we explained how the calibration was done under the effectiveness subsection.

Similarly, the utilities could not be verified from the sources. Please clarify how these values were calculated from the published sources, and if unpublished data can not be presented, be clear about this. Furthermore, the approach to utilities meant the improvements in quality of

life had the least impact for women aged 50-54 (general population utility was lowest at this age), which coincides with the median age of breast cancer diagnosis in Jordan (51). The authors should consider how the utility by age may have affected the effectiveness of the strategies, and whether this was plausible, e.g., the cross-sectional nature of the utility estimates may not translate to utility over an individual's lifetime.

Response: Thank you for your comment. We used the EQ-VAS data from the Jordanian EQ-5D valuation study because there are no age-specific health utility data in Jordan. The valuation study included EQ-VAS data from 301 individuals, and we had age and gender data, so we used EQ-VAS data for around half of them, categorized by age groups. We did not account for the impact of screening on HRQoL. The main impact will come from utilities per breast cancer stage.

Please present the distribution of the model parameters used in the PSA in the appendix to make the analysis more reproducible.

Response: Thank you for your comment. We updated the inputs table to include the distribution of parameters that were used in the PSA

It would be good to describe the choice of willingness to pay threshold in the methods and why it was chosen. Lines 484-490 in the discussion were confusing given Scenario 3 was described as meeting both thresholds.

Response: Thank you for your comment. In Jordan, we do not have a national threshold. We have a cancer-specific threshold that primarily addresses antineoplastic assessments. The choice of the willingness to pay threshold and its rationale is now added to the methods part under the base case analysis section, page 19

I did want to note that the validation methods were very clearly stated and reasonable, though they might be easier to discuss following the main results or in the discussion when comparing to external sources.

Response: Thank you for your comment. The validation results are reported in the results part under the calibration results and model validation section, page 21.

I think extended dominance results can be a little tricky for readers. It might be better to present all ICERs versus current practice and then the extended dominance analysis

Response: Thank you for your comment. We agree with these comments and have updated the results per the reviewer's recommendation. Table 3 now include the base case results based on comparing each strategy with the current comparator

Please complete a CHEERS checklist as recommended by BMJOpen and make sure the paper aligns with these guidelines (e.g., state whether a health economic plan was written prior to the analysis)

Response: Thank you for your comment. We prepared a conceptualization document before implementing the model, including a health economics plan. We updated the structure of the methods section to match the CHEERS checklist. We also attached a completed CHEERs check list with the revised submission

Overall, I think the paper has the potential to be a good publication and thank the authors for their submission.

VERSION 2 - REVIEW

**Reviewer**	**1**
**Name**	**Dack, Kyle**
**Affiliation**	**University of Bristol, MRC Integrative Epidemiology Unit**
**Date**	**04-May-2026**
**COI**	

I appreciate the substantial revisions made by the authors. My comments and queries were mostly related to clarifying the reporting of the methods and results and adherence to CHEER. The reporting checklist has now been fully applied and I have no further suggestions.

**Reviewer**	**2**
**Name**	**Auguste, Peter**
**Affiliation**	**University of Warwick Warwick Medical School**
**Date**	**21-May-2026**
**COI**	

Many thanks to the authors for careful consideration of the peer reviewers' feedback and for making the necessary changes to the manuscript, which has improved.

However, there are a couple of minor corrections required:

Table 8 provides the full incremental cost-effectiveness ratios (ICERs), which excludes dominated (dominated and extendedly dominated) strategies. In the title states …after elimination of extended dominance strategies but the correct terminology would be 'dominant' strategies, as this would include those that are strictly dominated and those that are extendedly dominated.

Table 9 reports the summary of the sensitivity analysis results. Three parameters are being included in the sensitivity analysis but there are several strategies. I don't think I have come across a sensitivity analysis where more than one strategy is being compared, i.e., using a

fully incremental analysis. It is more common, to undertake a pairwise comparison, e.g., Strategy 1 versus Comparator, and report the results in the form of a tornado diagram showing, which parameters are the most influential. Hence, in this case there will be several tornado diagrams because of the number of strategies. I raise this because of the 'extended dominance' result for Strategy 5, which would not have been possible in a pairwise comparison.

Apart from these minor points, the authors have made the necessary changes to the manuscript.

Reviewer **3**

Name **Huxley, Nicola**

Affiliation **Monash University, Centre for Health Economics, Monash Business School**

Date **30-May-2026**

COI

Thank you for the edit of this paper, there was much more detail than the previous version. The methods seem appropriate, but I do still have some suggestions with regards the write-up and presentation of methods and results.

Introduction: Thank you for the extra detail, I think it has helped contextualise the paper. In general, I still think there is confusion in the paper between availability/opportunity for screening (e.g., factors influencing clinicians offering screening) and uptake of screening (e.g., factors influencing patients when screening is available). The paper focuses on the first, but referring to "low attendance" covers both drivers. Just something to be clear about throughout the paper.

Methods: Thank you for trying to align with the CHEERs checklist. Note this checklist applies to the whole paper, and therefore you may address items in other sections to avoid duplication and repetition in the methods section. Table 1 of the paper might be better as supplemental table, to allow you to bring more important tables into the main body of the paper. For example, a lot of the parameters are only reported in supplementary tables, and are not referenced in the text. It would be preferable to have key parameter values stated in the main body of the paper, and at minimum, please ensure all supplemental tables are referenced in the text where they are described (i.e., not in the results section).

Health Economics Analysis Plan: this is usually a statement on whether a plan existed prior to the analysis. If not, please state so. If it did, please link to this plan/add to attachments, and make sure to comment when the analysis differed from the plan.

Sections Study population to Study perspective: Check these sections for duplication and repetition with the Introduction, and limit to how the analysis differs to anything you said there.

Time horizon: By lifetime, it would be good to clarify maximum age, and the resulting total years in the base case.

Identification, measurement, and valuation of outcomes/Effectiveness: add references to the first sentence for "literature"

Rationale and description of the model: it is unusual to have the model description after the inputs. The structure of the model informs the choice of inputs (e.g., utilities have to be chosen to align with various health states). Suggest moving model structure description to earlier in the methods.

Results. As with methods, it would be better to have main result tables and figures in the main body of the paper, than in the supplementary appendix.

Study parameters: this should be in the methods. Study parameters only need to be in the results if they had to be calculated during the analysis. Also I note there is an additional cost of invitation for screening in organised setting, but would there be other set-up/ongoing costs of introducing organised screening that should be considered?

Model's outcomes: effectiveness results, line 479: "Around 85 to 153 additional breast cancer cases", a reminder of population/cohort size and underlying breast cancer prevalence might be useful to interpret these numbers. Also be clear whether results are at cohort/population or individual patient level (e.g., reporting number of cases by cohort, but number or QALYs by patient).

Sensitivity analysis

DSA, line 517: presumably strategy 3 under the 100% attendance SA would no longer be defined as 50-65, biennial, 50% attendance?

PSA, line 524: should "overleaping" be "overlapping"?

Discussion, line 582: This <5% screening attendance needs to be in the introduction. This is the driver for why an organised screening programme is important. Also, lines 583-584, if only aiming for a maximum attendance of 40%, why does the model look at 50%?

References: I haven't checked all of these, as I found multiple errors or lack of information in the first 20 I checked. Please go through and ensure all information, particularly authors are correct. For webpages, give exact webpage titles, publishers/authors, publication date and access date for the data. If the source was a pdf, please manually add the authors, title, year, publisher etc.

Some examples:

3: this is not a reference (and annual is spelled incorrectly)

7: not a reference

8: Austrian, not Austrain (I assume)

14: Check authorship is correct (Comission E)

16: I couldn't find this reference, please check author and add any web address etc.

18: check first author, the version I found was first authored by Tala Hamadeh

19: specific webpage, access dates for this please

Supplemental figures: on all graphs be clear whether you are reporting total or incremental costs and QALYs. And consider alternative shapes/line styles for arms- multiple blues are difficult to differentiate. BMJOpen is unlikely to redraw these for a supplemental appendix, so they need to be clear for the reader.

For Figure 2 (the PSA cost-effectiveness plane), it may be worth presenting the incremental plane versus the comparator (current practice), to see which quadrants the simulations lie in. Currently this plot is hard to decipher. Also state that this is the PSA results and who may simulations were run.

Figure 3, it may be worth presenting a cost-effectiveness acceptability frontier (CEAF) in addition to the CEAC, to address the overlapping lines. Note this is different from the cost-effectiveness frontier presented in Figure 4.

Figure 4 appears to be the cost-effectiveness frontier with the costs and QALY axes swapped (usually we interpret these that steeper lines are the ones we don't want, because costs are on the y-axis and QALYs on the x-axis). Consider also reporting the ICERs versus the next best comparators next to the lines to explain the lines between the points, and include a footnote to explain what this figure shows. If Figure 4 is based on the base case deterministic results, I would consider moving it earlier and discuss it alongside the base case results table. This is a good visual way to show dominance and extended dominance and complements the base case results table nicely. (if it is based on the probabilistic results, this should also be stated, but I think is probably less informative unless there is a significant difference between the probabilistic and deterministic results)

Overall, I think the edits have improved this paper

VERSION 2 - AUTHOR RESPONSE

Reviewer: 1
Dr. Kyle Dack, University of Bristol
Comments to the Author:
I appreciate the substantial revisions made by the authors. My comments and queries were mostly related to clarifying the reporting of the methods and results and adherence to CHEER. The reporting checklist has now been fully applied and I have no further suggestions.

Response: Thank you.

Reviewer: 2
Mr. Peter Auguste, University of Warwick Warwick Medical School
Comments to the Author:
Many thanks to the authors for careful consideration of the peer reviewers' feedback and for making the necessary changes to the manuscript, which has improved.

However, there are a couple of minor corrections required:

Table 8 provides the full incremental cost-effectiveness ratios (ICERs), which excludes dominated (dominated and extendedly dominated) strategies. In the title states …after elimination of extended dominance strategies but the correct terminology would be 'dominant' strategies, as this would include those that are strictly dominated and those that are extendedly dominated.
Response: Thank you for this comment. We adjusted the title of Table 8 in the supplementary materials (page 15).

Table 9 reports the summary of the sensitivity analysis results. Three parameters are being included in the sensitivity analysis but there are several strategies. I don't think I have come across a sensitivity analysis where more than one strategy is being compared, i.e., using a fully incremental analysis. It is more common, to undertake a pairwise comparison, e.g., Strategy 1 versus Comparator, and report the results in the form of a tornado diagram showing, which parameters are the most influential. Hence, in this case there will be several tornado diagrams because of the number of strategies. I raise this because of the 'extended dominance' result for Strategy 5, which would not have been possible in a pairwise comparison.

Response: Thank you for this comment. In this analysis, reported in Table 9, we intended to show how the ranking changes as input parameters vary. Strategy three remained in rank 1 despite changes in input parameters.

Apart from these minor points, the authors have made the necessary changes to the manuscript.
Response: Thank you.

Reviewer: 3
Dr. Nicola Huxley, Monash University
Comments to the Author:
Thank you for the edit of this paper, there was much more detail than the previous version. The methods seem appropriate, but I do still have some suggestions with regards the write-up and presentation of methods and results.

Introduction: Thank you for the extra detail, I think it has helped contextualise the paper. In general, I still think there is confusion in the paper between availability/opportunity for screening (e.g., factors influencing clinicians offering screening) and uptake of screening (e.g., factors influencing patients when screening is available). The paper focuses on the first,

but referring to “low attendance” covers both drivers. Just something to be clear about throughout the paper.

Response: Thank you for your comment. Opportunistic screening is related to clinician referrals and is not systematic or organized. This reflects that breast cancer screening in Jordan is not systematic, and we wanted to compare systematic strategies with this current situation. Under this opportunistic scenario, we have a low attendance rate. In this case, by the attendance rate, we mean the number of women who attended screening in Jordan among those eligible, in a context where attendance is triggered by clinical referrals or by breast cancer screening awareness campaigns. In addition, in this case of opportunistic screening, there is no regular invitation for women to attend screening.
When we say a low attendance rate, we mean a low actual attendance among women under the current unsystematic screening situation in Jordan. I think both drivers are related, and the assumption is that if we have a systematic screening (organized), the attendance rate is expected to increase, as reliance on referral by clinicians in the current situation did not lead to a high attendance rate. We added a definition for attendance rate under the comparator opportunistic screening strategy as follows: The attendance rate for the opportunistic strategy reflects the participation rate of the eligible women population.

Methods: Thank you for trying to align with the CHEERs checklist. Note this checklist applies to the whole paper, and therefore you may address items in other sections to avoid duplication and repetition in the methods section. Table 1 of the paper might be better as supplemental table, to allow you to bring more important tables into the main body of the paper. For example, a lot of the parameters are only reported in supplementary tables, and are not referenced in the text. It would be preferable to have key parameter values stated in the main body of the paper, and at minimum, please ensure all supplemental tables are referenced in the text where they are described (i.e., not in the results section).

Response: Thank you for your comment. The input parameters are distributed across three tables: 1, 2, and 3. Previously, they were part of the main text, but the Journal requested, during the technical check, either to shorten these tables or to move them to the supplementary material, especially since Table 1 is long. We could not shorten them, so they were kept in the supplementary.
Tables 1, 2, and 3 are referenced in the study parameters subsection of the results, page 22. Previously, the input parameters were listed in the methods section, but according to the CHEERS checklist, they should be reported in the results section. Nevertheless, we referenced the supplementary tables, where the inputs were described, primarily in the methods section.

Health Economics Analysis Plan: this is usually a statement on whether a plan existed prior to the analysis. If not, please state so. If it did, please link to this plan/add to attachments, and make sure to comment when the analysis differed from the plan.

Response : Thank you for your comment .We had an analysis plan described in the project’s conceptualization document. We complied with the plan regarding the model structure and type ,the need for calibration ,and the sources of inputs .We adjusted the Health Economics Analysis Plan section of the study’s methods section to reflect its linkage to the project’s conceptualization

internal document .Page 15 ,lines :309-310.

Sections Study population to Study perspective :Check these sections for duplication and repetition with the Introduction ,and limit to how the analysis differs to anything you said there.

Response : Thank you for your comment .We removed the perspective from the introduction and kept it under its specific section in the methods.

Time horizon :By lifetime ,it would be good to clarify maximum age ,and the resulting total years in the base case.

Response : Thank you for your comment .The maximum age was 100 years ,yielding a total of 61 elapsed years in the base case .We adjusted the manuscript to reflect this modification . Page 17 ,lines :348-349.

Identification ,measurement ,and valuation of outcomes/Effectiveness :add references to the first sentence for "literature"

Response : Thank you for your comment .References were added.

Rationale and description of the model :it is unusual to have the model description after the inputs .The structure of the model informs the choice of inputs (e.g., utilities have to be chosen to align with various health states .(Suggest moving model structure description to earlier in the methods.

Response : Thank you for your comment .Figure 1 shows the model structure. In the revised manuscript, we referred to it in the analysis plan section under the methods.

Results. As with methods, it would be better to have main result tables and figures in the main body of the paper, than in the supplementary appendix.

Response : Thank you for your comment .There is a limited number of acceptable figures and tables in the main manuscript ,and we could not include all the main results tables and figures in the main document.

Study parameters :this should be in the methods .Study parameters only need to be in the results if they had to be calculated during the analysis .Also I note there is an additional cost of invitation for screening in organised setting ,but would there be other set-up/ongoing costs of introducing organised screening that should be considered?

Response : Thank you for your comment .According to the CHEERS 2 checklist ,the input parameters subsection is the first one in the results section .It is item 22 in the CHEERS checklist ,which states "report all analytical inputs (such as values ,ranges ,references) including uncertainty or distributional assumption" [1].

Regarding the additional cost ,we included the estimated cost of running an organized program ,including coordination ,sending invites ,and administration .The Jordan breast cancer program is fully established and already has the required human resources to implement this role in the

future if required .Also ,due to the fragmentation of the Jordanian health care system and the ability of different organizations to provide screening ,we only estimated the cost of coordination and administration of organized screening rather than investing in infrastructure to be able to implement organized screening ,so this is a resource management rather than the need for new
investment.

Model's outcomes**:** effectiveness results, line 479: "Around 85 to 153 additional breast cancer cases", a reminder of population/cohort size and underlying breast cancer prevalence might be useful to interpret these numbers. Also be clear whether results are at cohort/population or individual patient level (e.g., reporting number of cases by cohort, but number or QALYs by patient).

Response: Thank you for your comment. Regarding QALYs, it is the average QALY per simulated individual in a cohort. On the other hand, our model also predicts the number of breast cancer cases for each screening strategy. We calculated the prevalence-to-incidence ratio, which was 3.4% for observed cases and 3.7% for model-predicted cases. The number of predicted cases is adjusted to the women's population size per age group for 2018. In addition, in the introduction, we reported the number of breast cancer cases registered in Jordan in 2018 and the cumulative total from 1996 to 2018.

Sensitivity analysis
DSA, line 517: presumably strategy 3 under the 100% attendance SA would no longer be defined as 50-65, biennial, 50% attendance?
Response: Thank you for your comment. We agree with this comment, and the definition has been corrected.

PSA, line 524: should "overleaping" be "overlapping"?
Response: Thank you for your comment. The spelling mistake was corrected.

Discussion, line 582: This <5% screening attendance needs to be in the introduction. This is the driver for why an organised screening programme is important. Also, lines 583-584, if only aiming for a maximum attendance of 40%, why does the model look at 50%?

Response: Thank you for your comment. We corrected the targeted screening uptake to 50%. Regarding the attendance rate, which is the participation rate for opportunistic screening, it was less than 5% when we initially started the project; however, in recent years, there has been an increase in the participation rate, reaching 13% according to the Jordan Breast Cancer Program's recent data. Despite the increase to 13%, participation remains low. Several references report different percentages, reaching 17%. Therefore, we kept the most recent reference and removed this percentage. However, when we started this project, the percentage was less than 5%.

References: I haven't checked all of these, as I found multiple errors or lack of information in the first 20 I checked. Please go through and ensure all information, particularly authors are correct. For webpages, give exact webpage titles, publishers/authors, publication date and access date for the data. If the source was a pdf, please manually add the authors, title, year,

publisher etc.
Some examples:
3: this is not a reference (and annual is spelled incorrectly): This is our published national cancer registry numbers document for 2018, reference corrected
7: not a reference: corrected
8: Austrian, not Austrain (I assume): corrected
14: Check authorship is correct (Comission E): corrected
16: I couldn't find this reference, please check author and add any web address etc: corrected
18: check first author, the version I found was first authored by Tala Hamadeh: corrected
19: specific webpage, access dates for this please: corrected

Response: Thank you for your comment. All references have been reviewed and adjusted when needed.

Supplemental figures: on all graphs be clear whether you are reporting total or incremental costs and QALYs. And consider **alternative shapes/line styles for arms- multiple blues are difficult to differentiate**. BMJOpen is unlikely to redraw these for a supplemental appendix, so they need to be clear for the reader.

Response: Thank you for your comment. We plotted total cost and total QALYs; the graphs were adjusted accordingly. Alternative colors and shapes were also used to improve the clarity of the graphs.

For Figure 2 (the PSA cost-effectiveness plane), it may be worth presenting the incremental plane versus the comparator (current practice), to see which quadrants the simulations lie in. Currently this plot is hard to decipher. Also state that this is the PSA results and who may simulations were run.

Response: Thank you for your comment. We adjusted Figure 2 according to recommendations.

Figure 3, it may be worth presenting a cost-effectiveness acceptability frontier (CEAF) in addition to the CEAC, to address the overlapping lines. Note this is different from the cost-effectiveness frontier presented in Figure 4.

Response: Thank you for your comment. We added Figure 5 to the supplementary materials, which presents the cost-effectiveness acceptability frontier (CEAF).

Figure 4 appears to be the cost-effectiveness frontier with the costs and QALY axes swapped (usually we interpret these that steeper lines are the ones we don't want, because costs are on the y-axis and QALYs on the x-axis). Consider also reporting the ICERs versus the next best comparators next to the lines to explain the lines between the points, and include a footnote to explain what this figure shows. If Figure 4 is based on the base case deterministic results, I would consider moving it earlier and discuss it alongside the base case results table. This is a good visual way to show dominance and extended dominance and complements the base case results table nicely. (if it is based on the probabilistic results, this should also be stated, but I

think is probably less informative unless there is a significant difference between the probabilistic and deterministic results)

Response: Thank you for your comment. We adjusted Figure 4 according to recommendations. Yes, Figure 4 is based on the deterministic results, and we added this information to the figure's footnote.

References

1. Husereau D, Drummond M, Augustovski F, de Bekker-Grob E, Briggs AH, Carswell C, Caulley L, Chaiyakunapruk N, Greenberg D, Loder E, Mauskopf J, Mullins CD, Petrou S, Pwu RF, Staniszewska S. Consolidated Health Economic Evaluation Reporting Standards (CHEERS) 2022 Explanation and Elaboration: A Report of the ISPOR CHEERS II Good Practices Task Force. Value Health. 2022 Jan;25(1):10-31. doi: 10.1016/j.jval.2021.10.008. Erratum in: Value Health. 2022 Jun;25(6):1060. doi: 10.1016/j.jval.2022.03.002. PMID: 35031088.
