# Supplementary material for: Cost-effectiveness of organised versus opportunistic mammography breast cancer screening in low-resourced country
Source: BMJ Open. 2026 Aug 17;16(8):e108486. doi: 10.1136/bmjopen-2025-108486 (PMC13504863; doi:10.1136/bmjopen-2025-108486)
Supplement: Supplementary file 2 [file bmjopen-16-8-s001.docx]

**Supplementary material**

**Table (1). Disease natural history input parameters and screening effectiveness measures**

| **Progression** |  | **Annual transition probability** | **Mean value** | **Probability distribution** | **Reference** |
| --- | --- | --- | --- | --- | --- |
| *From* | *To* |  |  |  |  |
| **Age-specific progression from no diagnosed cancer to pre-clinical undetected DCIS** | | | | | |
|  |  | Age |  |  |  |
| No cancer | Undetected pre-clinical DCIS | 20-29 | 0.0000051 | Uniform | Calibration |
|  |  | 30-39 | 0.0000052 | Uniform | Calibration |
|  |  | 40--49 | 0.0002332 | Uniform | Calibration |
|  |  | 50-59 | 0.0002520 | Uniform | Calibration |
|  |  | 60-69 | 0.0006216 | Uniform | Calibration |
|  |  | 70-79 | 0.0009425 | Uniform | Calibration |
| **Age-specific regression from pre-clinical undetected DCIS to no cancer** | | | | | |
| Undetected pre-clinical DCIS | No cancer | 20-29 | 0.1182452 | Uniform | Calibration, (1) |
|  |  | 30-39 | 0.0915809 | Uniform | Calibration,(1) |
|  |  | 40--49 | 0.0928232 | Uniform | Calibration,(1) |
|  |  | 50-59 | 0.1028741 | Uniform | Calibration,(1) |
|  |  | 60-69 | 0.1080423 | Uniform | Calibration,(1) |
|  |  | 70-79 | 0.0978581 | Uniform | Calibration,(1) |
| **Age-specific progression from no cancer directly to pre-clinical undetected stage 1** | | | | | |
| No diagnosed cancer | Undetected pre-clinical stage 1 | 20-29 | 0.0000164 | Uniform | Calibration |
|  |  | 30-39 | 0.0002078 | Uniform | Calibration |
|  |  | 40--49 | 0.0009340 | Uniform | Calibration |
|  |  | 50-59 | 0.0015535 | Uniform | Calibration |
|  |  | 60-69 | 0.0015761 | Uniform | Calibration |
|  |  | 70-79 | 0.0021014 | Uniform | Calibration |
| **Age-specific progression from pre-clinical undetected DCIS to clinically detected DCIS** | | | | | |
| Pre-clinical undetected DCIS | Clinically detected DCIS | 20-29 | 0.0060780 | Uniform | Calibration |
|  |  | 30-39 | 0.0048472 | Uniform | Calibration |
|  |  | 40--49 | 0.0052080 | Uniform | Calibration |
|  |  | 50-59 | 0.0048589 | Uniform | Calibration |
|  |  | 60-69 | 0.0049873 | Uniform | Calibration |
|  |  | 70-79 | 0.0046692 | Uniform | Calibration |
| **Age-specific progression from pre-clinical undetected DCIS to pre-clinical undetected S1** | | | | | |
| Pre-clinical undetected DCIS | Pre-clinical undetected stage 1 | 20-29 | 0.1653165 | Uniform | Calibration |
|  |  | 30-39 | 0.1607539 | Uniform | Calibration |
|  |  | 40--49 | 0.1652055 | Uniform | Calibration |
|  |  | 50-59 | 0.1668402 | Uniform | Calibration |
|  |  | 60-69 | 0.1881147 | Uniform | Calibration |
|  |  | 70-79 | 0.1521774 | Uniform | Calibration |
|  | **Age-specific progression from pre-clinical undetected stage 1 to clinically detected stage 1** | | | | |
| Pre-clinical undetected stage 1 | Clinically detected stage 1 | 20-29 | 0.1149081 | Uniform | Calibration |
|  |  | 30-39 | 0.1161357 | Uniform | Calibration |
|  |  | 40--49 | 0.1328884 | Uniform | Calibration |
|  |  | 50-59 | 0.1189713 | Uniform | Calibration |
|  |  | 60-69 | 0.1320364 | Uniform | Calibration |
|  |  | 70-79 | 0.1048156 | Uniform | Calibration |
|  |  |  | 0.1099260 | Uniform | Calibration |
| **Age-specific progression from pre-clinical undetected stage 1 to pre-clinical undetected stage 2** | | | | | |
| Pre-clinical undetected stage 1 | Pre-clinical undetected stage 2 | 20-29 | 0.2659073 | Uniform | Calibration |
|  |  | 30-39 | 0.2547626 | Uniform | Calibration |
|  |  | 40--49 | 0.2313292 | Uniform | Calibration |
|  |  | 50-59 | 0.2116004 | Uniform | Calibration |
|  |  | 60-69 | 0.2153881 | Uniform | Calibration |
|  |  | 70-79 | 0.2066330 | Uniform | Calibration |
| **Age-specific progression from pre-clinical undetected stage 2 to clinically detected stage 2** | | | | | |
| Pre-clinical undetected stage 2 | Clinically detected stage 2 | 20-29 | 0.3662840 | Uniform | Calibration |
|  |  | 30-39 | 0.3693557 | Uniform | Calibration |
|  |  | 40--49 | 0.3633407 | Uniform | Calibration |
|  |  | 50-59 | 0.3329623 | Uniform | Calibration |
|  |  | 60-69 | 0.3840415 | Uniform | Calibration |
|  |  | 70-79 | 0.3770078 | Uniform | Calibration |
| **Age-specific progression from pre-clinical undetected stage 2 to pre-clinical undetected stage 3** | | | | | |
| Pre-clinical undetected stage 2 | Pre-clinical undetected stage 3 | 20-29 | 0.5125006 | Uniform | Calibration |
|  |  | 30-39 | 0.4838946 | Uniform | Calibration |
|  |  | 40--49 | 0.5135281 | Uniform | Calibration |
|  |  | 50-59 | 0.5433237 | Uniform | Calibration |
|  |  | 60-69 | 0.4847949 | Uniform | Calibration |
|  |  | 70-79 | 0.5069104 | Uniform | Calibration |
| **Age-specific progression from pre-clinical undetected stage 3 to clinically detected stage 3** | | | | | |
| Pre-clinical undetected stage 3 | Clinically detected stage 3 | 20-29 | 0.6600436 | Uniform | Calibration |
|  |  | 30-39 | 0.6627043 | Uniform | Calibration |
|  |  | 40--49 | 0.6423216 | Uniform | Calibration |
|  |  | 50-59 | 0.6541934 | Uniform | Calibration |
|  |  | 60-69 | 0.6441197 | Uniform | Calibration |
|  |  | 70-79 | 0.6598447 | Uniform | Calibration |
| **Age-specific progression from pre-clinical undetected stage 3 to pre-clinical undetected stage 4** | | | | | |
| Pre-clinical undetected stage 3 | Pre-clinical undetected stage 4 | 20-29 | 0.4850646 | Uniform | Calibration |
|  |  | 30-39 | 0.5426952 | Uniform | Calibration |
|  |  | 40--49 | 0.4672436 | Uniform | Calibration |
|  |  | 50-59 | 0.5485930 | Uniform | Calibration |
|  |  | 60-69 | 0.5474432 | Uniform | Calibration |
|  |  | 70-79 | 0.5707488 | Uniform | Calibration |
| **Age-specific progression from pre-clinical undetected stage 4 to clinically detected stage 4** | | | | | |
| Pre-clinical undetected stage 4 | Clinically detected stage 4 | 20-29 | 0.8154406 | Uniform | Calibration |
|  |  | 30-39 | 0.8054625 | Uniform | Calibration |
|  |  | 40--49 | 0.8589653 | Uniform | Calibration |
|  |  | 50-59 | 0.8401493 | Uniform | Calibration |
|  |  | 60-69 | 0.8377131 | Uniform | Calibration |
|  |  | 70-79 | 0.8009503 | Uniform | Calibration |
| **Disease and stage specific 5 years survival rate** | | | | | |
| **Probability of death from breast cancer stage 1** |  |  | 0.0283700 | Beta (α**=**2, β=114) | (2) |
| **Probability of death from breast cancer stage 1** |  |  | 0.0461724 | Beta (α=0.60, β=33) | (2) |
| **Probability of death from breast cancer stage 1** |  |  | 0.1578165 | Beta (α=5, β=33) | (2) |
| **Probability of death from breast cancer stage 1** |  |  | 0.3240114 | Beta (α11=, β=25) | (2) |
| **Screening effectiveness** | | | | | |
| **Mammography sensitivity** | | | | | |
|  | | 40-44 | 72% (0.69-0.74) | Beta (α=878, β=343) | (3) |
|  |  | 45-49 | 76% (0.74-0.78) | Beta (α=1589, β=502) | (3) |
|  |  | 50-54 | 80% (0.72-0.86) | Beta (α=95, β=24) | (3) |
|  |  | 55-59 | 84% (0.78-0.88) | Beta (α=181, β=36) | (3) |
|  |  | 60-64 | 87% (0.81-0.91) | Beta (α=154, β=23) | (3) |
|  |  | 65-69 | 83% (0.77-0.89) | Beta (α=121, β=25) | (3) |
|  |  | 70-74 | 84% (0.82-0.85) | Beta (α=1823, β=350) | (3) |
|  |  | 75+ | 85% (0.84-0.86) | Beta (α=2631, β=461) | (3) |
| **Mammography specificity** | | | | | |
|  | | 40-44 | 89% (0.88-0.89) | Beta(α=460103, β=56287) | (3) |
|  |  | 45-49 | 90% (0.89-0.90) | Beta(α=570214, β=66186) | (3) |
|  |  | 50-54 | 90% (0.89-0.90) | Beta(α=,34573  β=3842) | (3) |
|  |  | 55-59 | 90% (0.90-0.91) | Beta(α=38755, β=4163) | (3) |
|  |  | 60-64 | 91% (0.90-0.91) | Beta(α=30057, β=3009) | (3) |
|  |  | 65-69 | 92% (0.91-0.92) | Beta(α=24359, β=2263) | (3) |
|  |  | 70-74 | 93% (0.92-0.93) | Beta(α=283644, β=21022) | (3) |
|  |  | 75+ | 94% (0.93-0.94) | Beta(α=336560, β=23013) | (3) |
| **Screening attendance rate current situation** | | | | | |
|  | | All ages | 3.5% | Beta(α=10, β=313) | JBCP unpublished data,(4) |

**Table (2). Heath State Utilities**

| **Health State** | **Mean Utility (SE)** | **Probability distribution** | **Reference** |
| --- | --- | --- | --- |
| Utility general population for age group 40_44 | 0.816 (0.027) | Beta (α=160, β=36) | Jordan EQ-5D-3L valuation study (5) |
| Utility general population for age group 45_49 | 0.804(0.027) | Beta (α=165, β=41) | Jordan EQ-5D-3L valuation study (5) |
| Utility general population for age group 50_54 | 0.696(0.027) | Beta (α=192, β=84) | Jordan EQ-5D-3L valuation study (5) |
| Utility general population for age group 55_59 | 0.794(0.027) | Beta (α=169, β=44) | Jordan EQ-5D-3L valuation study (5) |
| Utility general population for age group 60_64 | 0.758(0.027) | Beta (α=181, β=58) | Jordan EQ-5D-3L valuation study (5) |
| Utility general population for age group 65+ | 0.743(0.027) | Beta (α=185, β=64) | Jordan EQ-5D-3L valuation study (5) |
| Utility of breast cancer stage 1 | 0.680(0.004) | Beta (α=6411, β=3017) | (6) |
| Utility of breast cancer stage 2 | 0.610(0.004) | Beta (α=6588, β=4021) | (6) |
| Utility of breast cancer stage 3 | 0.560 (0.046) | Beta (α=62, β=49) | (6) |
| Utility of breast cancer stage 4 | 0.420 (0.071) | Beta (α=19, β=28) | (6) |

SE: standard error

**Table (3). Aggregate costs (in 2022 US$) for screening, diagnosis, and treatment**

| **Resource** | | **Costs (in 2022 US$)** | | **Probability distribution** | | **Reference** |
| --- | --- | --- | --- | --- | --- | --- |
| **Screening** | | | | | | |
| Organized mean screening cost per woman (includes mammography, ultrasound, physician reading, and recall (10%), cost of invitation) | | 71.00 (53-89) | | Gamma (α=61, β=1.1) | | (7) |
| Opportunistic mean screening Cost per woman (includes mammography, ultrasound, physician reading, and recall (10%)) | | 68.00 (51-85) | | Gamma (α=60, β=1.2) | | (7) |
| **Treatment cost** | | | | | | |
| **Initial cancer treatment cost (surgery, radiotherapy, and medications)** | | | | | | |
| **Stage 0** | 10,104 | | 7,578-12,630 | | Gamma (α=61, β=164) | (7-9) |
| **Stage 1** | 15,994 | | 11,995-19,992 | | Gamma (α=61, β=260) | (7-9) |
| **Stage 2** | 23,029 | | 17,272-28,787 | | Gamma (α=61, β=375) | (7-9) |
| **Stage 3** | 29,664 | | 22,248-37,080 | | Gamma (α=61, β=483) | (7-9) |
| **Stage 4** | 43,380 | | 32,535-54,225 | | Gamma (α=61, β=706) | (7-9) |
| **Follow up cost (annual costs)** | | | | | | |
| **Stage 0** | 686 | | 514-857 | | Gamma (α=61, β=11) | (8) |
| **Stage 1** | 282 | | 212-353 | | Gamma (α=61, β=5) | (8) |
| **Stage 2** | 263 | | 197-329 | | Gamma (α=61, β=4) | (8) |
| **Stage 3** | 445 | | 334-556 | | Gamma (α=61, β=7) | (8) |
| **Stage 4** | 1164 | | 837-1455 | | Gamma (α=61, β=19) | (8) |

**Table (4). Model’s validation results**

| **Validation Target** | **Observed**  **(national cancer registry data 2018)** | **Model predictions** |
| --- | --- | --- |
| Breast cancer deaths | 342 | 347 |
| Median age at diagnosis (years) | 51 | 50.6 |
| DCIS | 2% | 2% |
| Stage 2 | 30% | 26.2% |
| Stage 3 | 20% | 18.5% |
| Stage 4 | 13% | 13.9% |

**Table (5). Breast cancer predicted cases and deaths over a lifetime per screening strategy**

|  |  | Comparator | Strategy 1 | Strategy 2 | Strategy 3 | Strategy 4 | Strategy 5 | Strategy 6 |
| --- | --- | --- | --- | --- | --- | --- | --- | --- |
| DCIS | Clinically detected | 4 | 3 | 3 | 4 | 3 | 3 | 3 |
|  | Screen detected cases | 19 | 71 | 83 | 60 | 104 | 79 | 117 |
| Stage 1 | Clinically detected | 357 | 275 | 274 | 302 | 248 | 288 | 227 |
|  | Screen detected cases | 72 | 306 | 308 | 229 | 388 | 274 | 450 |
| Stage 2 | Clinically detected | 235 | 168 | 163 | 187 | 142 | 174 | 126 |
|  | Screen detected cases | 16 | 54 | 56 | 44 | 68 | 52 | 75 |
| Stage 3 | Clinically detected | 169 | 110 | 112 | 130 | 91 | 119 | 77 |
|  | Screen detected cases | 6 | 21 | 20 | 18 | 26 | 21 | 26 |
| Stage 4 | Clinically detected | 125 | 75 | 73 | 89 | 58 | 80 | 48 |
|  | Screen detected cases | 4 | 9 | 11 | 9 | 11 | 11 | 11 |
|  | Total predicted cases | 1007 | 1092 | 1103 | 1072 | 1139 | 1101 | 1160 |
|  | Deaths | 265 | 223 | 226 | 239 | 211 | 233 | 199 |

**Table (6). Base case results based on comparing each strategy with the current comparator**

| **Strategies** | **LY undisc** | **QALY undisc** | **Cost undisc (US$)** | **LY disc** | **QALY disc** | **Cost disc (US$)** | **Inc LY** | **Inc dis QALY** | **Inc dis Cost**  **(US$)** | **ICER (US$/LY)** | **ICUR (US$/QALY)** |
| --- | --- | --- | --- | --- | --- | --- | --- | --- | --- | --- | --- |
| Comparator  40-70, annual, attendance rate 3.5% | 36.619 | 27.914 | 1755 | 22.062 | 16.980 | 880 | 0 | 0 | 0 | 0 | 0 |
| Strategy1  40-60, biennial, attendance rate 50% | 36.667 | 27.952 | 1974 | 22.082 | 16.996 | 1078 | 0.02 | 0.016 | 199 | 9,867 | 12,302 |
| Strategy2  50-65, annual,  attendance rate 50% | 36.670 | 27.957 | 2083 | 22.081 | 16.998 | 1102 | 0.019 | 0.018 | 223 | 11,948 | 12,644 |
| Strategy3  50-65, biennial, attendance rate 50% | 36.653 | 27.944 | 1866 | 22.075 | 16.993 | 964 | 0.013 | 0.012 | 84 | 6,678 | 6,734 |
| Strategy4  40-70, biennial, attendance rate 50% | 36.683 | 27.967 | 2062 | 22.087 | 17.002 | 1133 | 0.025 | 0.022 | 253 | 10,222 | 11,590 |
| Strategy5  50-70 biennial, attendance rate 50% | 36.661 | 27.948 | 1918 | 22.077 | 16.993 | 994 | 0.015 | 0.013 | 114 | 7,764 | 8,986 |
| Strategy6  40-49 annual, 50-70 biennial, attendance rate 50% | 36.694 | 27.973 | 2220 | 22.092 | 17.004 | 1275 | 0.03 | 0.024 | 395 | 13,170 | 16,387 |

Strategies ranked from lowest cost and QALYs to highest cost and QALYs. LY: life years, dis: discounted, undisc: undiscounted, QALY: quality adjusted life years, Inc: incremental, ICER: incremental cost effectiveness ratio. ICUR: incremental cost utility ratio. ICER and ICUR are calculated using discounted outcomes.

**Table (7). Base case results, comparing each intervention with the following effective strategy**

| **Strategies** | **LY disc** | **QALY disc** | **Cost disc (US$)** | **Inc LY** | **Inc QALY** | **Inc Cost**  **(US$)** | **ICER (US$/LY)** | **ICUR (US$/QALY)** |
| --- | --- | --- | --- | --- | --- | --- | --- | --- |
| Comparator  40-70, annual, attendance rate 3.5% | 22.062 | 16.980 | 880 | 0 | 0 | 0 | 0 | 0 |
| Strategy3  50-65, biennial, attendance rate 50% | 22.075 | 16.993 | 964 | 0.013 | 0.012 | 84 | 6,462 | 6,734 |
| Strategy5  50-70, biennial, attendance rate 50% | 22.077 | 16.993 | 994 | 0.002 | 0.000 | 30 | 14,103 | 118,295 |
| Strategy1  40-60, biennial, attendance rate 50% | 22.082 | 16.996 | 1078 | 0.005 | 0.003 | 84 | 15,563 | 24,549 |
| Strategy2  50-65, annual, attendance rate 50% | 22.081 | 16.998 | 1102 | -0.001 | 0.001 | 24 | Dominated | 16,430 |
| Strategy4  40-70, biennial, attendance rate 50% | 22.087 | 17.002 | 1133 | 0.006 | 0.004 | 31 | 4,981 | 7,212 |
| Startegy6  40-49 annual, 50-70 biennial, attendance rate 50% | 22.092 | 17.004 | 1274 | 0.005 | 0.002 | 142 | 27,106 | 62,418 |

*Ranked from lowest cost and QALYs to highest cost and QALYs. LY: life years, dis: discounted, QALY: quality adjusted life years, Inc: incremental, ICER: incremental cost effectiveness ratio. ICUR: incremental cost utility ratio.

**Table (8). Recalculation of ICERs after elimination of dominant strategies**

| **Strategies** | **QALY disc** | **Cost disc (US$)** | **Comparison** | **Inc QALY** | **Inc Cost**  **(US$)** | **ICUR (US$/QALY)** |
| --- | --- | --- | --- | --- | --- | --- |
| **Comparator (C)** | 16.980 | 880 |  | 0 | 0 | 0 |
| Strategy 3 | 16.993 | 964 | 3 vs C | 0.012 | 89 | 6,734 |
| Strategy 5 | 16.993 | 994 | 5 vs 3 | 0.000 | 30 | 118,295 (dominated) |
| Strategy 1 | 16.996 | 1078 | 1 vs 5 | 0.003 | 84 | 24,549 |
| Strategy 2 | 16.998 | 1102 | 2 vs 1 | 0.001 | 24 | 16,430 |
| Strategy 4 | 17.002 | 1133 | 4 vs 2 | 0.004 | 31 | 7,212 |
| Strategy 6 | 17.004 | 1275 | 6 vs 4 | 0.002 | 142 | 62,418 |
| After excluding strategy 5 (dominated by strategy 3) | | | | | | |
| Comparator | 16.980 | 880 |  | 0 | 0 | 0 |
| Strategy 3 | 16.993 | 964 | 3 vs C | 0.012 | 84 | 6,734 |
| Strategy 1 | 16.996 | 1078 | 1 vs 3 | 0.004 | 115 | 31,051  (extended dominance) |
| Strategy 2 | 16.998 | 1102 | 2 vs 1 | 0.001 | 24 | 16,430 |
| Strategy 4 | 17.002 | 1133 | 4 vs 2 | 0.004 | 31 | 7,212 |
| Strategy 6 | 17.004 | 1275 | 6 vs 4 | 0.002 | 142 | 62,418 |
| After excluding extended dominance strategy | | | | | | |
| **Comparator** | 16.980 | 875 |  | 0 | 0 | 0 |
| Strategy 3 | 16.993 | 964 | 3 vs C | 0.012 | 84 | 6,734 |
| Strategy 2 | 16.998 | 1102 | 2 vs 3 | 0.005 | 139 | 26,910  (extended dominance) |
| Strategy 4 | 17.002 | 1133 | 4 vs 2 | 0.004 | 31 | 7,212 |
| Strategy 6 | 17.004 | 1275 | 6 vs 4 | 0.002 | 142 | 62,418 |
| Final remaining strategies | | | | | | |
| **Comparator** | 16.980 | 875 |  | 0 | 0 | 0 |
| Strategy 3 | 16.993 | 964 | 3 vs C | 0.012 | 84 | 6,734 |
| Strategy 4 | 17.002 | 1133 | 4 vs 3 | 0.009 | 169 | 18,024 |
| Strategy 6 | 17.004 | 1275 | 6 vs 3 | 0.002 | 142 | 62,418 |

Comparator (current situation) 40-70, annual attendance rate 3.5%, Strategy1: 40-60, biennial, attendance rate 50%, Strategy2: 50-65, annual, attendance rate 50%, Strategy3: 50-65, biennial, attendance rate 50%, Strategy4: 40-70, biennial, attendance rate 50%, Strategy5: 50-70 biennial, attendance rate 50%, Strategy6: 40-49 annual, 50-70 biennial, attendance rate 50%.

**Table (9). Summary of sensitivity analysis results**

|  | **ICUR (US$/QALY)** | | | | | | |  |
| --- | --- | --- | --- | --- | --- | --- | --- | --- |
| **Strategy** | **Base case** | **Varied parameter** | | | | | |  |
|  |  | **Costs of organized screening package ($US)** | | **Discount rate** | | **Effect measure**  **(sensitivity, specificity)** | |  |
|  |  | Lower | Upper | 0% | 6% | Lower | Upper |  |
| **Comparator** | Ref | Ref | Ref | Ref | Ref | Ref | Ref |  |
| **Strategy1** | 24,549  *Rank 3* | 17,964  *Rank 3* | 31,133  *Rank 3* | 16,846  *Rank 3* | 108,684  *Rank 3* | 23,742  *Rank 3* | *24,899*  *Rank 3* |  |
| **Strategy2** | 16,430  *Rank 4* | 11,768  *Rank 4* | 21,091  *Rank 4* | 19,807  *Rank 4* | Dominating scenario 1  More benefit and less cost  *Rank 4* | 15,914  *Rank 4* | *17,617*  *Rank 4* |  |
| **Strategy3** | 6,734  *Rank 1* | 4,689.59  *Rank 1* | 9,533  *Rank 1* | 3,677  *Rank 1* | Dominating scenario 5  More benefit and less cost  *Rank 2* | 7,673  *Rank 1* | *6,760*  *Rank 1* |  |
| **Strategy4** | 7,212  Extended dominance  *Rank 5* | 4,825  Extended dominance  *Rank 5* | 9,600 Extended dominance  *Rank 5* | Dominating scenario 2  More benefit and less cost  *Rank 5* | 28,936  *Rank 5* | 7,696  Extended dominance  *Rank 5* | 6,761  Extended dominance  *Rank 5* |  |
| **Strategy5** | 118,295  *Rank 2* | 82,660  *Rank 2* | 153,930  *Rank 2* | 10,958  *Rank 2* | 15,422  *Rank 1* | 160,357  *Rank 2* | *90,060*  *Rank 2* |  |
|  |  |  |  |  |  |  |  |  |
| **Strategy6** | 62,418  *Rank 6* | 45,604  *Rank 6* | 79,232  *Rank 6* | 23,560  *Rank 6* | 976,756  *Rank 6* | 60,324  *Rank 6* | *64,022*  *Rank 6* |  |
|  |  |  |  |  |  |  |  |  |

Comparator (current situation) 40-70, annual, attendance rate 3.5%, Strategy1 40-60, biennial, attendance rate 50%, Strategy2 50-65, annual, attendance rate 50%, Strategy3 50-65, biennial, attendance rate 50%, Strategy4 40-70, biennial, attendance rate 50%, Strategy5, 50-70, biennial, attendance rate 50%, Strategy6 40-49 annual, 50-70 biennial, attendance rate 50%.

**Table (10). Summary of sensitivity analysis results for screening attendance rate**

| 10% attendance rate for proposed stratgies |  |  |  |  |
| --- | --- | --- | --- | --- |
| Strategies | LY disc | QALY disc | Cost disc | ICUR |
| Strategy 5 | 22.062 | 16.980 | 873 |  |
| Comparator | 22.062 | 16.980 | 880 | 52,667 |
| Strategy 1 | 22.063 | 16.981 | 890 | 12,169 |
| Strategy 3 | 22.061 | 16.981 | 867 | (131,543) |
| Strategy 4 | 22.065 | 16.984 | 899 | 11,929 |
| Strategy 2 | 22.065 | 16.984 | 890 | (33,266) |
| Strategy 6 | 22.067 | 16.984 | 925 | 245,644 |
| 100% attendance rate for proposed stratgies | |  |  |  |
| Strategies | LY disc | QALY disc | Cost disc | ICUR |
| Comparator | 22.062 | 16.980 | 880 |  |
| Straetegy 6 | 22.067 | 16.984 | 925 | 11,000 |
| Strategy 3 | 22.084 | 17.001 | 1101 | 10,670 |
| Strategy 5 | 22.087 | 17.002 | 1165 | 61,843 |
| Strategy 2 | 22.088 | 17.004 | 1397 | 108,432 |
| strategy 1 | 22.095 | 17.007 | 1336 | (21,948) |
| Strategy 4 | 22.102 | 17.014 | 1452 | 15,918 |
| 10% attendance rate comparator |  |  |  |  |
| Strategies | LY disc | QALY disc | Cost disc | ICUR |
| Comparator | 22.071 | 16.987 | 946 |  |
| Strategy 3 | 22.075 | 16.993 | 964 | 3,418 |
| Strategy 5 | 22.077 | 16.993 | 994 | 118,295 |
| Strategy 1 | 22.082 | 16.996 | 1078 | 24,549 |
| Strategy 2 | 22.081 | 16.998 | 1102 | 16,430 |
| Strategy 4 | 22.087 | 17.002 | 1133 | 7,212 |
| Strategy 6 | 22.092 | 17.004 | 1275 | 62,418 |
| 100% attendance rate comparator |  |  |  |  |
| Stratgies | LY disc | QALY disc | Cost disc | ICUR |
| Strategy 3 | 22.075 | 16.993 | 964 |  |
| Strategy 5 | 22.077 | 16.993 | 994 | 118,295 |
| Strategy 1 | 22.082 | 16.996 | 1078 | 24,549 |
| Strategy 2 | 22.081 | 16.998 | 1102 | 16,430 |
| Strategy 4 | 22.087 | 17.002 | 1133 | 7,212 |
| Strategy 6 | 22.092 | 17.004 | 1275 | 62,418 |
| Comparator | 22.109 | 17.018 | 2035 | 54,051 |

Comparator (current situation) 40-70, annual attendance rate 3.5%, Strategy1 40-60, biennial, attendance rate 50%, Strategy2 50-65, annual, attendance rate 50%, Strategy3 50-65, biennial, attendance rate 50%, Strategy4 40-70, biennial, attendance rate 50%, Strategy5, 50-70 biennial, attendance rate 50%, Strategy6 40-49 annual, 50-70 biennial, attendance rate 50%.

**Table (11). Deterministic versus probabilistic cost effectiveness results per strategy**

| **Strategies** | **Deterministic**  **ICUR (US$/QALY)** | **Probabilistic**  **ICUR (US$/QALY)** |
| --- | --- | --- |
| Comparator  40-70, annual, attendance rate 3.5% | 0 |  |
| Strategy3  50-65, biennial, attendance rate 50% | 6,734 | 7,504.63 |
| Strategy5  50-70, biennial, attendance rate 50% | 118,295 | Dominated by strategy 3 |
| Strategy1  40-60, biennial, attendance rate 50% | 24,549 | 6,244.49 |
| Strategy2  50-65, annual, attendance rate 50% | 16,430 | Dominated by strategy 1 |
| Strategy4  40-70, biennial, attendance rate 50% | 7,212 | 1,190.58 |
| Startegy6  40-49 annual, 50-70 biennial, attendance rate 50% | 62,418 | Dominated by strategy 4 |

*Ranked from lowest cost and QALYs to highest cost and QALYs. LY: life years, dis: discounted, QALY: quality adjusted life years, Inc: incremental, ICER: incremental cost effectiveness ratio. ICUR: incremental cost utility ratio.

Predicted versus Observed Breast Cancer Cases per Age Group

|  | Observed | Predicted |
| --- | --- | --- |
| 20 -24 | 4 | 2.4 |
| 25 -29 | 19 | 18.3 |
| 30 - 34 | 65 | 44.1 |
| 35 - 39 | 108 | 101 |
| 40 - 44 | 194 | 186.7 |
| 45 - 49 | 228 | 227.7 |
| 50 - 54 | 213 | 217.7 |
| 55 - 59 | 183 | 174.5 |
| 60 - 64 | 125 | 128.1 |
| 65 - 69 | 123 | 120.4 |
| 70 - 74 | 91 | 88.3 |
| 75 - 79 | 71 | 59.5 |
| 80 - 84 | 22 | 24.1 |
| 85+ | 16 | 20.5 |

**Figure (1). Model calibration results (validation)**

Total Mean Cost ($US)

| Total Mean Effectiveness (QALYs) | Strategy1 |
| --- | --- |
| 16.8755 | 1165.75 |
| 16.6518 | 1066.01 |
| 16.7764 | 1067.04 |
| 16.5438 | 1129.15 |
| 16.7945 | 1022.47 |
| 16.2224 | 1136.82 |
| 16.4456 | 1185.47 |
| 16.8394 | 1281.55 |
| 16.6353 | 1042.33 |
| 16.7082 | 1056.87 |
| 16.6857 | 1204.18 |
| 16.6247 | 1235.12 |
| 16.3114 | 1168.88 |
| 16.9023 | 1091.18 |
| 16.5781 | 1090.5 |
| 16.912 | 1085.45 |
| 16.721 | 1045.79 |
| 16.5582 | 1070.52 |
| 16.6827 | 1163.92 |
| 17.2152 | 945.751 |
| 16.7071 | 1241.48 |
| 16.9783 | 1257.7 |
| 16.3994 | 1162.44 |
| 16.2905 | 1158.17 |
| 16.6344 | 1146.58 |
| 16.3499 | 994.745 |
| 17.1457 | 1108.92 |
| 16.9384 | 926.587 |
| 16.5662 | 1022.66 |
| 16.6976 | 1034.96 |
| 16.4872 | 909.257 |
| 16.5882 | 1078.23 |
| 17.1444 | 1006.97 |
| 16.2561 | 1135.61 |
| 16.2395 | 1035.06 |
| 16.4382 | 1097.17 |
| 16.4652 | 991.631 |
| 16.8481 | 1209.37 |
| 16.6898 | 1071.7 |
| 17.0767 | 1119.31 |
| 16.513 | 980.549 |
| 16.4728 | 1346.89 |
| 16.958 | 1109.15 |
| 16.4796 | 1098.56 |
| 16.7636 | 1028.75 |
| 16.6724 | 1061.12 |
| 16.562 | 1213.9 |
| 16.6868 | 1261.04 |
| 16.7039 | 1228.77 |
| 16.9698 | 1099.76 |
| 16.9442 | 1097.87 |
| 16.6159 | 1098.51 |
| 16.7946 | 1039.85 |
| 16.5972 | 1071.76 |
| 16.6082 | 1163.99 |
| 16.4588 | 1103.08 |
| 16.7296 | 1131.52 |
| 16.8639 | 1029.94 |
| 16.5389 | 1106.98 |
| 16.5861 | 950.516 |
| 16.365 | 994.345 |
| 16.6568 | 1059.69 |
| 16.9276 | 1012.04 |
| 16.3589 | 1084.97 |
| 17.1604 | 1265.55 |
| 16.1565 | 1097.58 |
| 16.1982 | 1313.96 |
| 16.8669 | 1166 |
| 16.7216 | 1051.31 |
| 16.8673 | 1036.01 |
| 16.4375 | 1039.48 |
| 16.2513 | 1057.56 |
| 16.4523 | 1332.5 |
| 16.6058 | 956.27 |
| 17.0791 | 994.892 |
| 16.565 | 1192.6 |
| 16.5685 | 1145.47 |
| 16.8339 | 1251.34 |
| 16.5506 | 1025.68 |
| 16.6582 | 1203.67 |
| 16.8654 | 974.354 |
| 16.3384 | 1157.32 |
| 16.9576 | 911.277 |
| 16.1646 | 1010.1 |
| 16.5973 | 1126.58 |
| 16.6872 | 1198.76 |
| 16.6571 | 1123.02 |
| 16.5774 | 1039.67 |
| 16.607 | 1128.99 |
| 16.607 | 1162.6 |
| 16.2131 | 1136.46 |
| 16.3231 | 1180.38 |
| 16.743 | 1076.98 |
| 16.7861 | 1088.61 |
| 16.4874 | 1087.45 |
| 16.5353 | 985.969 |
| 16.6247 | 1247 |
| 16.5196 | 1012.76 |
| 16.98 | 1208.23 |
| 16.7511 | 1090.09 |
| 16.7145 | 1058.23 |
| 16.5125 | 1136.89 |
| 16.8428 | 1086.26 |
| 16.3086 | 1002.42 |
| 16.0805 | 1037.7 |
| 16.6666 | 1122.24 |
| 16.7483 | 1048.29 |
| 16.2442 | 1238.93 |
| 16.7364 | 1088.69 |
| 16.335 | 1113.07 |
| 16.9792 | 1061.83 |
| 16.5896 | 1161.25 |
| 16.7414 | 949.113 |
| 16.4893 | 1024.46 |
| 16.9566 | 1050.39 |
| 16.4751 | 1208.67 |
| 16.3597 | 1195.77 |
| 16.4588 | 1151.12 |
| 16.6411 | 1129.89 |
| 16.8508 | 1132.95 |
| 16.6722 | 1242.25 |
| 16.8035 | 1066.57 |
| 16.8188 | 992.754 |
| 16.3795 | 1094.37 |
| 16.5018 | 1111.48 |
| 16.7861 | 1288.93 |
| 16.5484 | 1139.12 |
| 16.9328 | 1068.26 |
| 16.5555 | 1291.85 |
| 16.7487 | 1134.89 |
| 16.6591 | 1002.92 |
| 16.5989 | 1031.54 |
| 16.4348 | 1113.55 |
| 16.5225 | 1213.41 |
| 16.7935 | 1084.01 |
| 16.1276 | 1245.54 |
| 16.6547 | 959.248 |
| 16.4289 | 1134.4 |
| 16.7594 | 1156.43 |
| 16.6543 | 1205.4 |
| 16.3763 | 967.328 |
| 16.4357 | 1160.9 |
| 16.9261 | 1107.32 |
| 16.9083 | 1023.86 |
| 16.077 | 1039.59 |
| 16.9406 | 977.536 |
| 16.9822 | 1127.48 |
| 16.6373 | 1120.5 |
| 16.744 | 1094.54 |
| 16.4006 | 1119.69 |
| 16.6322 | 1150.92 |
| 16.7029 | 1041.17 |
| 16.9132 | 1241.39 |
| 16.3055 | 1044.47 |
| 16.3018 | 945.873 |
| 16.6036 | 1159.4 |
| 16.657 | 1131.1 |
| 17.0709 | 1105.95 |
| 16.3777 | 1096.95 |
| 16.5994 | 1022.55 |
| 16.8969 | 1043.95 |
| 16.9278 | 1101.41 |
| 16.7058 | 1005.25 |
| 16.5515 | 1045.86 |
| 16.3608 | 1034.56 |
| 16.4119 | 1114.29 |
| 16.1822 | 1037.97 |
| 16.9805 | 1042.08 |
| 17.2731 | 1113.85 |
| 16.9278 | 1290.21 |
| 16.4347 | 1025 |
| 16.3062 | 1074.59 |
| 16.2849 | 1188.89 |
| 16.8251 | 1123.57 |
| 16.6314 | 1054.86 |
| 16.813 | 1091.38 |
| 16.521 | 1023.72 |
| 16.8977 | 1047.23 |
| 16.9403 | 1088.74 |
| 16.4834 | 1102.6 |
| 16.6861 | 1176.08 |
| 16.8406 | 1101.68 |
| 16.9726 | 1066.25 |
| 16.8842 | 1234.25 |
| 16.2323 | 1120.97 |
| 16.8633 | 1024.82 |
| 17.0535 | 1121.12 |
| 16.8707 | 905.835 |
| 17.2485 | 1061.05 |
| 16.4708 | 1154.56 |
| 16.8891 | 1232.5 |
| 16.2867 | 1089.03 |
| 16.6409 | 1145.02 |
| 16.5652 | 1021.24 |
| 16.5107 | 1023.99 |
| 16.6273 | 1181.17 |
| 16.4565 | 1112.1 |
| 16.1486 | 1210.75 |
| 16.7994 | 1188.58 |
| 16.5978 | 1062.82 |

| Total Mean Effectiveness (QALYs) | Strategy 2 |
| --- | --- |
| 16.5985 | 1168.63 |
| 16.5881 | 1068.63 |
| 16.3582 | 1230.86 |
| 16.338 | 1003.67 |
| 15.9026 | 1090.87 |
| 16.944 | 1112.5 |
| 16.2663 | 1236.03 |
| 16.546 | 1098.19 |
| 16.5104 | 1160.54 |
| 16.7834 | 1144.19 |
| 16.6153 | 1308.24 |
| 16.6354 | 1252.96 |
| 16.6498 | 1257.38 |
| 16.5532 | 1175.4 |
| 16.7575 | 1119.8 |
| 16.5399 | 1161.31 |
| 17.308 | 1213.37 |
| 16.4847 | 1068.23 |
| 17.0468 | 1175.46 |
| 16.5255 | 1326.9 |
| 16.5941 | 1166.47 |
| 16.5843 | 1103.81 |
| 16.8635 | 1038.51 |
| 16.4806 | 1254.22 |
| 16.6908 | 1213.29 |
| 16.3024 | 1144 |
| 16.4993 | 1099.83 |
| 16.8546 | 1169.72 |
| 16.7478 | 1137.52 |
| 16.7308 | 1111.49 |
| 17.0732 | 1258.76 |
| 16.5939 | 1065.88 |
| 16.3625 | 1236.74 |
| 16.6407 | 1238.34 |
| 16.5964 | 1047.83 |
| 16.9258 | 1162.13 |
| 16.516 | 1121.11 |
| 16.2703 | 1158.42 |
| 16.8143 | 1284.91 |
| 16.8928 | 1137.05 |
| 16.9902 | 1262.55 |
| 16.5997 | 1375.45 |
| 16.3834 | 1371.44 |
| 16.8572 | 1080.16 |
| 16.8045 | 1020.51 |
| 16.7129 | 1279.79 |
| 16.6825 | 1278.81 |
| 15.9543 | 1158.9 |
| 16.3987 | 1174.56 |
| 16.9295 | 1123.96 |
| 16.3084 | 1080.92 |
| 16.8427 | 1233.75 |
| 16.4261 | 994.443 |
| 16.5769 | 1128.27 |
| 16.2111 | 1082.2 |
| 16.3586 | 1076.46 |
| 16.397 | 1039.6 |
| 16.6848 | 994.074 |
| 16.4535 | 1135.19 |
| 16.54 | 1116.93 |
| 16.51 | 1137.26 |
| 16.4843 | 1107.2 |
| 16.8124 | 1242.15 |
| 16.678 | 1090.77 |
| 17.0312 | 1243.92 |
| 16.5026 | 1118.1 |
| 16.6769 | 1127.05 |
| 16.4886 | 992.865 |
| 16.4244 | 1210.05 |
| 16.438 | 1152.17 |
| 16.9481 | 1210.42 |
| 16.5613 | 1134.49 |
| 16.8013 | 1020.06 |
| 16.5133 | 1218.26 |
| 16.7693 | 1042.09 |
| 16.3259 | 1004.12 |
| 16.4543 | 1194.48 |
| 16.3379 | 1206.47 |
| 16.6977 | 1077.78 |
| 16.6292 | 1111.76 |
| 16.621 | 1172.83 |
| 16.2332 | 1155.87 |
| 16.6489 | 1223.83 |
| 16.4955 | 1149.72 |
| 16.6646 | 1225.02 |
| 16.7313 | 1142.4 |
| 16.3627 | 973.751 |
| 16.6602 | 1193.64 |
| 16.7939 | 1149.23 |
| 16.961 | 1085.77 |
| 17.0759 | 1086.36 |
| 16.4866 | 1253.94 |
| 16.4379 | 1045.92 |
| 16.644 | 989.565 |
| 16.668 | 1177.73 |
| 16.7087 | 1087.56 |
| 16.6525 | 1052.36 |
| 16.4099 | 1211.41 |
| 16.5644 | 1024.89 |
| 16.283 | 1169.8 |
| 16.3005 | 1122.49 |
| 17.131 | 1138.18 |
| 16.8899 | 1187.87 |
| 16.2904 | 1099.23 |
| 16.4046 | 1175.47 |
| 16.6313 | 1078.24 |
| 17.0835 | 1076.36 |
| 17.1048 | 1117.52 |
| 16.6938 | 1092.95 |
| 16.5629 | 1129.14 |
| 16.6568 | 1323.93 |
| 16.3122 | 1066.84 |
| 16.545 | 1050.92 |
| 16.7371 | 1061.86 |
| 16.0647 | 1043.85 |
| 16.4508 | 1120.48 |
| 17.0256 | 1100.93 |
| 16.5101 | 1196.85 |
| 16.5184 | 1019.56 |
| 16.652 | 1094.79 |
| 16.6995 | 1173.64 |
| 16.2941 | 1173.78 |
| 16.2709 | 1145.9 |
| 16.6891 | 1098.8 |
| 16.7816 | 1286.27 |
| 16.9812 | 1246.65 |
| 16.6794 | 1046.25 |
| 16.4531 | 1091.4 |
| 16.8687 | 1254.42 |
| 16.3926 | 1061.44 |
| 16.4752 | 1095.16 |
| 16.8227 | 1061.29 |
| 16.2115 | 1195.93 |
| 16.0486 | 1076.03 |
| 16.3948 | 1217.58 |
| 17.2029 | 1136.48 |
| 16.5854 | 1109.26 |
| 16.6715 | 1255.1 |
| 16.7402 | 1078.12 |
| 16.8871 | 1089.01 |
| 16.3607 | 1064.6 |
| 16.9838 | 1277.04 |
| 16.3831 | 1229.74 |
| 16.9888 | 1105.88 |
| 16.5306 | 1224.78 |
| 16.2355 | 1105.61 |
| 16.5488 | 1137.2 |
| 16.6714 | 1089.08 |
| 16.8644 | 1041.8 |
| 16.5556 | 1026.05 |
| 16.7191 | 1007.92 |
| 16.52 | 1120.1 |
| 16.3241 | 1089.37 |
| 16.8304 | 1111.05 |
| 16.2773 | 1227.64 |
| 16.6634 | 1083.86 |
| 17.0491 | 1151.84 |
| 16.5489 | 1225.51 |
| 16.6503 | 1071.93 |
| 16.9442 | 1155.9 |
| 16.5955 | 1154.31 |
| 16.6702 | 1027.64 |
| 16.6022 | 1109.28 |
| 17.1396 | 1085.11 |
| 16.7105 | 1050.35 |
| 16.717 | 1171.39 |
| 16.6604 | 992.632 |
| 16.4445 | 1128.81 |
| 16.4198 | 1251.64 |
| 16.5857 | 1161.49 |
| 16.7407 | 1189.85 |
| 16.4328 | 1031.97 |
| 16.7349 | 1193.88 |
| 16.8527 | 1112.05 |
| 16.4532 | 1235.42 |
| 16.4113 | 1114.66 |
| 16.6006 | 1187.25 |
| 16.4028 | 1148.42 |
| 16.8046 | 1156.49 |
| 16.7964 | 1171.58 |
| 16.4912 | 1067.19 |
| 16.4016 | 1078.34 |
| 16.8308 | 1179.43 |
| 16.3241 | 1252.04 |
| 16.7525 | 1099.84 |
| 16.5012 | 1175.25 |
| 16.3816 | 1053.8 |
| 16.2866 | 1175.68 |
| 16.7086 | 1108.59 |
| 16.5601 | 1011.37 |
| 16.6839 | 1223.59 |
| 16.6162 | 1117.62 |
| 16.6695 | 1092.21 |
| 17.0326 | 1200.82 |
| 16.0286 | 983.189 |
| 17.0355 | 1010.38 |
| 16.6306 | 1052.73 |
| 16.9278 | 1075.69 |
| 16.593 | 1177.21 |
| 16.5659 | 970.96 |

| Total Mean Effectiveness (QALYs) | Strategy 3 |
| --- | --- |
| 16.6949 | 1130.51 |
| 16.3303 | 1019.81 |
| 16.9301 | 964.785 |
| 16.4598 | 998.954 |
| 16.5203 | 947.499 |
| 16.4863 | 1135.82 |
| 16.8103 | 1004 |
| 16.5725 | 1082.56 |
| 17.0119 | 906.819 |
| 16.9415 | 982.585 |
| 16.1262 | 985.898 |
| 16.2738 | 892.389 |
| 16.646 | 884.379 |
| 16.8949 | 1002.09 |
| 16.7566 | 1014.85 |
| 16.9128 | 970.506 |
| 17.0434 | 1090.65 |
| 16.6885 | 1082.89 |
| 16.7084 | 998.571 |
| 16.5101 | 1042.59 |
| 16.5138 | 1158.07 |
| 16.5178 | 1029.65 |
| 16.4942 | 986.398 |
| 16.6644 | 969.03 |
| 16.6431 | 988.848 |
| 16.7127 | 1107.52 |
| 16.4992 | 1081.45 |
| 16.8082 | 962.953 |
| 16.1482 | 935.681 |
| 16.1744 | 1073.03 |
| 16.5478 | 1117.08 |
| 16.8256 | 1050.38 |
| 16.5589 | 1027.29 |
| 16.8274 | 1040.71 |
| 17.058 | 861.759 |
| 16.4048 | 936.432 |
| 16.585 | 968.222 |
| 16.8734 | 1263.22 |
| 16.5697 | 1122.24 |
| 16.5598 | 938.782 |
| 16.9283 | 1045.56 |
| 16.588 | 1023.75 |
| 16.7132 | 1165.93 |
| 16.8672 | 908.768 |
| 16.4672 | 1093.73 |
| 16.2579 | 1039.79 |
| 16.287 | 942.811 |
| 16.3821 | 943.099 |
| 16.5224 | 1135.52 |
| 16.125 | 989.593 |
| 16.3454 | 978.538 |
| 16.893 | 1086.61 |
| 16.4779 | 1188.76 |
| 16.1548 | 936.687 |
| 16.2991 | 1021.49 |
| 16.6029 | 1125.06 |
| 16.6018 | 1029.43 |
| 15.7339 | 957.241 |
| 16.7206 | 1041.97 |
| 16.8846 | 1091.95 |
| 16.3818 | 1055.96 |
| 16.854 | 909.989 |
| 16.8392 | 1091.55 |
| 16.6849 | 963.301 |
| 16.1502 | 867.707 |
| 16.6934 | 1046 |
| 16.5319 | 849.336 |
| 16.3313 | 1046.66 |
| 16.8358 | 984.804 |
| 16.6168 | 1036.48 |
| 16.687 | 1011.67 |
| 16.3679 | 907.746 |
| 16.2986 | 1062.23 |
| 16.4893 | 962.268 |
| 16.6136 | 938.613 |
| 16.9868 | 979.379 |
| 17.0282 | 1004.61 |
| 16.5109 | 911.525 |
| 17.1049 | 950.491 |
| 16.6139 | 982.216 |
| 17.0037 | 903.591 |
| 16.5186 | 929.624 |
| 16.2583 | 1151.28 |
| 16.3941 | 1070.55 |
| 16.9145 | 988.992 |
| 16.8166 | 982.237 |
| 16.4035 | 1094.5 |
| 16.2716 | 907.292 |
| 16.2314 | 1000.35 |
| 16.464 | 1047.19 |
| 17.0691 | 1151.99 |
| 16.5505 | 1026.5 |
| 16.6854 | 1181.92 |
| 17.0482 | 1082.85 |
| 16.9484 | 1034.04 |
| 16.8815 | 909.141 |
| 16.6343 | 1215.26 |
| 16.1216 | 925.686 |
| 16.8885 | 1158.02 |
| 16.3851 | 1148.16 |
| 16.6252 | 1000.37 |
| 16.5931 | 1015.26 |
| 16.6905 | 1132.82 |
| 16.5787 | 915.57 |
| 16.4499 | 999.226 |
| 16.6227 | 939.76 |
| 16.2016 | 960.564 |
| 16.3018 | 931.429 |
| 16.5525 | 1005.63 |
| 16.5011 | 973.088 |
| 16.9471 | 1141.9 |
| 16.7229 | 1011.05 |
| 16.5565 | 1259.06 |
| 16.7566 | 1120.73 |
| 16.4684 | 996.462 |
| 16.8846 | 1043.52 |
| 16.3283 | 1049.93 |
| 16.2874 | 975.573 |
| 16.4266 | 983.69 |
| 16.7095 | 917.67 |
| 16.9185 | 859.684 |
| 16.7855 | 1032.62 |
| 16.5685 | 971.479 |
| 16.1117 | 953.529 |
| 16.6763 | 1153.64 |
| 16.4634 | 998.596 |
| 16.6715 | 982.785 |
| 16.3414 | 993.136 |
| 16.4833 | 1047.8 |
| 16.3607 | 1089.61 |
| 16.6594 | 1145.62 |
| 16.4164 | 1044.02 |
| 16.4342 | 1005.55 |
| 16.1508 | 1027.18 |
| 16.6148 | 975.091 |
| 16.5231 | 1107.06 |
| 16.4218 | 944.092 |
| 16.8191 | 952.991 |
| 16.8995 | 983.109 |
| 16.6012 | 1111.72 |
| 16.7035 | 1062.45 |
| 16.6342 | 1067.3 |
| 17.302 | 1008.1 |
| 16.202 | 981.751 |
| 16.6223 | 911.421 |
| 16.6567 | 893.058 |
| 16.6341 | 1060.85 |
| 16.6909 | 1055.96 |
| 16.4508 | 1084.01 |
| 16.3931 | 1098.59 |
| 16.9846 | 1019.29 |
| 16.5194 | 893.317 |
| 16.7038 | 1032.47 |
| 16.9363 | 1057.1 |
| 16.7944 | 1087.64 |
| 16.5483 | 1109.33 |
| 16.8782 | 979.842 |
| 16.1697 | 1036.63 |
| 16.7164 | 953.549 |
| 16.4028 | 948.59 |
| 16.4415 | 944.645 |
| 16.7619 | 929.583 |
| 16.7066 | 1007.52 |
| 16.3491 | 1129.25 |
| 17.1635 | 993.663 |
| 16.9699 | 928.208 |
| 16.6988 | 1046.63 |
| 16.8775 | 962.146 |
| 16.515 | 952.93 |
| 16.677 | 909.47 |
| 16.7432 | 991.375 |
| 16.7516 | 1022.91 |
| 16.4856 | 937.923 |
| 16.5849 | 876.621 |
| 16.6599 | 1123.41 |
| 16.5129 | 1074.6 |
| 16.6495 | 1028.72 |
| 16.8136 | 1066.11 |
| 16.9916 | 1007.57 |
| 16.9329 | 866.98 |
| 16.6113 | 856.004 |
| 16.3216 | 956.405 |
| 16.6536 | 959.136 |
| 16.0845 | 972.133 |
| 16.8829 | 1099.35 |
| 16.874 | 985.682 |
| 16.2201 | 904.516 |
| 16.6059 | 1106.16 |
| 16.4533 | 1000.2 |
| 16.4247 | 1119.18 |
| 16.558 | 892.32 |
| 16.7192 | 928.485 |
| 16.0374 | 992.518 |
| 16.514 | 907.338 |
| 16.8914 | 1004.48 |
| 16.7708 | 986.201 |
| 16.7249 | 1051.83 |
| 17.2439 | 935.298 |
| 16.5767 | 917.962 |
| 16.1809 | 946.043 |

| Total Mean Effectiveness (QALYs) | Strategy 4 |
| --- | --- |
| 17.3076 | 1202.34 |
| 16.5517 | 1173.44 |
| 16.7572 | 1108.96 |
| 16.533 | 1116.14 |
| 16.838 | 1268.83 |
| 16.7364 | 1183.38 |
| 16.6266 | 1148.83 |
| 16.6964 | 1275.92 |
| 15.9089 | 1156.38 |
| 17.0401 | 1108.01 |
| 17.2475 | 1204.52 |
| 16.8761 | 1244.57 |
| 16.5348 | 1197.78 |
| 16.5552 | 1141.14 |
| 16.5131 | 1237.99 |
| 16.6456 | 1208.26 |
| 16.5683 | 1152.68 |
| 16.3685 | 1340.29 |
| 16.5282 | 1127.98 |
| 16.6246 | 1247.65 |
| 16.4991 | 1132.27 |
| 16.5428 | 1117.62 |
| 16.8144 | 1227.67 |
| 16.4093 | 1198.04 |
| 16.8412 | 1113.11 |
| 16.667 | 1181.05 |
| 16.7064 | 1098.29 |
| 16.705 | 1195.21 |
| 16.5871 | 1175.18 |
| 16.3104 | 977.575 |
| 16.8736 | 1042.48 |
| 16.8543 | 1041.7 |
| 16.8444 | 1246.68 |
| 16.6929 | 1005.45 |
| 16.7076 | 1091.97 |
| 16.423 | 1199.55 |
| 16.5412 | 1142.76 |
| 16.8481 | 1197.47 |
| 16.6291 | 1173.61 |
| 16.8067 | 1051.52 |
| 15.9893 | 1110.68 |
| 16.2214 | 1094.89 |
| 16.4903 | 1348.62 |
| 16.5398 | 1084.89 |
| 16.1755 | 1262.07 |
| 16.5271 | 1197.67 |
| 16.435 | 1035.09 |
| 16.4589 | 1150.77 |
| 16.6258 | 1113.02 |
| 16.4057 | 1062.63 |
| 16.6354 | 1217.14 |
| 16.3841 | 1167.67 |
| 17.1309 | 1093.86 |
| 16.8345 | 1158.07 |
| 16.5416 | 1193.11 |
| 16.8431 | 1211.7 |
| 16.8086 | 1197.1 |
| 16.9903 | 1244.53 |
| 16.4291 | 1336.93 |
| 17.1585 | 1128.94 |
| 16.8555 | 1157.04 |
| 16.5989 | 1165.32 |
| 16.6272 | 1267.73 |
| 16.477 | 1149.46 |
| 16.2102 | 1141.62 |
| 16.9973 | 1200.88 |
| 16.1702 | 1149.18 |
| 16.6966 | 1054.25 |
| 16.5269 | 1154.72 |
| 16.7687 | 1113.64 |
| 16.5281 | 1051.91 |
| 16.9443 | 1073.29 |
| 16.4945 | 1241.22 |
| 16.2829 | 1055.06 |
| 16.6843 | 1151.66 |
| 16.6393 | 1287.3 |
| 16.6885 | 1101.43 |
| 16.3142 | 1066.89 |
| 16.6301 | 1076.59 |
| 17.0105 | 1041.47 |
| 16.5316 | 1102.37 |
| 16.4817 | 1359.16 |
| 16.6196 | 1049.49 |
| 16.5519 | 1287.45 |
| 16.8256 | 1159.78 |
| 16.5949 | 1114.63 |
| 16.2127 | 1289.62 |
| 16.6929 | 1143.77 |
| 16.8065 | 1209.28 |
| 16.1878 | 1182.46 |
| 16.7776 | 1179.09 |
| 16.9568 | 1137.02 |
| 16.3736 | 1087.56 |
| 16.1995 | 1181.71 |
| 16.5009 | 1108.47 |
| 16.6814 | 1145.05 |
| 16.9666 | 1048.28 |
| 16.9956 | 1276.43 |
| 16.6603 | 1304.89 |
| 16.6326 | 1310.8 |
| 16.2787 | 1133.57 |
| 16.6819 | 1211.9 |
| 16.6457 | 1070.37 |
| 16.9771 | 1170.8 |
| 16.5228 | 1116.53 |
| 16.7018 | 1053.14 |
| 16.4093 | 1266.55 |
| 16.94 | 1121.12 |
| 17.3167 | 1120.63 |
| 16.2943 | 1029.23 |
| 16.7806 | 1170.72 |
| 16.6489 | 1116.27 |
| 16.2657 | 1111.96 |
| 16.602 | 1156.96 |
| 16.5358 | 1063.31 |
| 16.8558 | 1264.76 |
| 17.1353 | 1051.39 |
| 16.6332 | 1250.06 |
| 16.6682 | 1195.2 |
| 15.8713 | 1103.44 |
| 16.4787 | 1112.92 |
| 16.6873 | 1170.95 |
| 16.71 | 1248.9 |
| 16.5199 | 1078.78 |
| 15.98 | 1127.87 |
| 16.659 | 1133.16 |
| 16.6324 | 1148.69 |
| 16.7895 | 1145.72 |
| 16.8453 | 1224.68 |
| 16.2506 | 1276.68 |
| 16.5027 | 991.969 |
| 16.611 | 1265.71 |
| 16.6962 | 1056.15 |
| 17.0669 | 1209.71 |
| 16.9164 | 1138.19 |
| 17.1294 | 1311.22 |
| 16.4723 | 1293.13 |
| 16.6094 | 1098.65 |
| 16.4757 | 1203.25 |
| 16.4876 | 989.53 |
| 16.9164 | 1153.13 |
| 16.7996 | 1321.89 |
| 16.1783 | 1180.37 |
| 16.7448 | 1227.51 |
| 16.799 | 1175.16 |
| 16.7918 | 1004.42 |
| 16.8988 | 1164.66 |
| 17.1726 | 1140.69 |
| 16.4273 | 1069.59 |
| 16.6058 | 1060.12 |
| 16.3496 | 1193.26 |
| 16.7769 | 1041.79 |
| 16.5075 | 1187.49 |
| 17.0047 | 1145 |
| 16.8186 | 1137.23 |
| 16.8096 | 1112.3 |
| 16.334 | 1075.49 |
| 16.2407 | 1135.18 |
| 16.4752 | 1171.5 |
| 16.5232 | 1306.92 |
| 16.7954 | 1236.92 |
| 17.0669 | 1315.46 |
| 16.5864 | 1152.74 |
| 16.8873 | 1425.54 |
| 16.5978 | 1090.16 |
| 16.6058 | 1130.3 |
| 16.3904 | 1201.07 |
| 16.3876 | 1123.68 |
| 16.7624 | 1332.6 |
| 16.6543 | 1179.71 |
| 16.8526 | 1217.65 |
| 16.6901 | 1110.92 |
| 16.5063 | 1100.94 |
| 16.5696 | 1033.43 |
| 16.6826 | 1265.17 |
| 16.6441 | 1050.39 |
| 16.7896 | 1256.33 |
| 17.0657 | 1109.99 |
| 16.7392 | 1039.94 |
| 16.068 | 1085.38 |
| 16.7718 | 1031.82 |
| 16.6227 | 1084.33 |
| 16.3678 | 1102.86 |
| 16.4924 | 1293.87 |
| 16.8493 | 1110.42 |
| 16.4894 | 1077.99 |
| 16.5269 | 1225.91 |
| 16.2351 | 960.072 |
| 16.5319 | 1090 |
| 16.7715 | 1083 |
| 17.2035 | 1213.15 |
| 16.8521 | 1076.94 |
| 17.2373 | 1102.25 |
| 16.1524 | 1027.88 |
| 17.1384 | 1114.64 |
| 16.697 | 1052.28 |
| 16.6919 | 1238.43 |
| 16.635 | 1190.74 |
| 16.3358 | 1077.41 |
| 16.6168 | 1132.53 |

| Total Mean Effectiveness (QALYs) | Strategy 5 |
| --- | --- |
| 16.863 | 1025.56 |
| 16.5648 | 964.214 |
| 16.7325 | 1090.71 |
| 16.4703 | 1010.99 |
| 16.8422 | 1040.83 |
| 16.0195 | 926.315 |
| 16.4274 | 1027.13 |
| 16.3539 | 1088.97 |
| 17.2857 | 1012.82 |
| 16.6447 | 959.104 |
| 16.823 | 1021.72 |
| 16.606 | 958.292 |
| 16.2467 | 1141.61 |
| 16.5515 | 1077.51 |
| 17.0707 | 964.954 |
| 16.6525 | 1098.08 |
| 16.2609 | 1119.81 |
| 16.4959 | 957.526 |
| 16.598 | 1059.91 |
| 16.4974 | 1008.74 |
| 16.4877 | 995.906 |
| 16.7203 | 1113.08 |
| 16.5228 | 973.102 |
| 17.1125 | 972.278 |
| 16.6129 | 1024.15 |
| 16.526 | 1049.45 |
| 16.7845 | 902.304 |
| 16.8865 | 1076.55 |
| 16.4283 | 1011.41 |
| 16.6367 | 1169.23 |
| 16.8003 | 998.432 |
| 16.3223 | 962.901 |
| 16.2481 | 968.578 |
| 16.5228 | 977.208 |
| 16.6781 | 1032.37 |
| 16.1845 | 1092.26 |
| 16.6769 | 1199.16 |
| 16.4313 | 1104.57 |
| 16.5141 | 1087.05 |
| 16.4513 | 1129.4 |
| 16.8918 | 1007.14 |
| 16.5114 | 1045.86 |
| 16.6143 | 1071.84 |
| 16.8268 | 977.457 |
| 16.8974 | 1043.75 |
| 16.6733 | 1085.32 |
| 16.5614 | 942.576 |
| 16.5963 | 1038.58 |
| 16.2892 | 1109.89 |
| 16.8827 | 1122.78 |
| 16.708 | 1056.99 |
| 16.2613 | 1116.95 |
| 16.7154 | 1074.5 |
| 16.3642 | 1044.49 |
| 17.0126 | 991.689 |
| 16.7637 | 1152.2 |
| 16.3785 | 1022.72 |
| 16.3316 | 997.784 |
| 16.4231 | 996.078 |
| 16.4481 | 937.038 |
| 16.3069 | 1000.23 |
| 17.0984 | 1176.21 |
| 16.6583 | 1107.69 |
| 16.9418 | 1094.38 |
| 16.4815 | 1003.3 |
| 16.6674 | 1076.74 |
| 16.6202 | 1116.2 |
| 16.2999 | 1092.83 |
| 16.1883 | 973.928 |
| 16.7873 | 984.46 |
| 16.4995 | 1041.59 |
| 16.5134 | 930.827 |
| 16.5299 | 1002.75 |
| 16.9527 | 984.473 |
| 16.4435 | 1085.59 |
| 16.5576 | 1022.7 |
| 16.4882 | 1122.95 |
| 16.7912 | 1047.8 |
| 16.5909 | 989.912 |
| 16.6774 | 1075.58 |
| 16.4362 | 1099.65 |
| 16.6597 | 1073.01 |
| 16.396 | 1016.89 |
| 16.8294 | 1015.17 |
| 16.5434 | 896.394 |
| 16.9935 | 874.827 |
| 17.0639 | 1094.88 |
| 16.6419 | 1064.78 |
| 17.0621 | 1069.42 |
| 15.9918 | 1157.38 |
| 16.6672 | 1022.83 |
| 16.8435 | 1090.58 |
| 16.6575 | 1091.62 |
| 16.3856 | 919.969 |
| 17.0156 | 1030.23 |
| 16.595 | 981.268 |
| 16.3238 | 950.992 |
| 16.7934 | 1101.6 |
| 16.9031 | 1067.54 |
| 16.3529 | 993.374 |
| 16.3524 | 923.904 |
| 16.5615 | 1074.49 |
| 16.344 | 922.15 |
| 16.5113 | 928.503 |
| 17.1198 | 1016.17 |
| 16.8422 | 1007.56 |
| 16.7509 | 1111.88 |
| 16.2744 | 916.323 |
| 16.4637 | 1231.17 |
| 16.6481 | 1157.64 |
| 16.5646 | 1025.24 |
| 17.0631 | 1002.06 |
| 16.9012 | 1055.43 |
| 16.5584 | 969.471 |
| 16.8267 | 872.574 |
| 16.8658 | 899.198 |
| 16.5475 | 1067.93 |
| 16.4289 | 1181.29 |
| 16.9817 | 952.349 |
| 16.3185 | 1068.9 |
| 17.022 | 940.121 |
| 16.4066 | 1098.05 |
| 16.3516 | 954.551 |
| 16.3848 | 982.193 |
| 17.0727 | 973.833 |
| 17.0056 | 1107.47 |
| 16.5947 | 1022.85 |
| 16.4977 | 1135.01 |
| 16.5449 | 1147.96 |
| 16.7134 | 1077.16 |
| 16.7309 | 958.472 |
| 17.1028 | 988.524 |
| 16.5911 | 1085.57 |
| 16.7644 | 923.965 |
| 16.6241 | 1064.61 |
| 16.8229 | 924.469 |
| 16.0859 | 1111.49 |
| 16.5285 | 981.406 |
| 16.7883 | 1083.87 |
| 16.7179 | 1076.52 |
| 16.6773 | 1003.1 |
| 16.8179 | 1023.36 |
| 16.3938 | 1117.43 |
| 16.9143 | 1129.07 |
| 16.7868 | 925.896 |
| 16.6211 | 1012.48 |
| 16.7329 | 1048.06 |
| 16.7521 | 1026.32 |
| 16.0942 | 1063.95 |
| 16.7237 | 935.376 |
| 16.6057 | 1170.55 |
| 16.6015 | 966.03 |
| 16.4817 | 981.823 |
| 16.6655 | 1021.26 |
| 16.6968 | 1310 |
| 16.2592 | 1045.1 |
| 16.3512 | 1042.82 |
| 16.3784 | 972.884 |
| 16.7673 | 1006.34 |
| 16.7806 | 1221.29 |
| 16.6746 | 991.623 |
| 16.5239 | 959.186 |
| 16.5614 | 1017.21 |
| 16.6202 | 929.778 |
| 16.7944 | 1066.07 |
| 16.6773 | 977.454 |
| 16.9514 | 958.615 |
| 16.5333 | 930.993 |
| 16.8848 | 1116.75 |
| 16.7613 | 1048.41 |
| 16.4694 | 1113.18 |
| 16.8564 | 893.511 |
| 17.0389 | 958.242 |
| 16.445 | 907.294 |
| 16.7587 | 951.87 |
| 16.8749 | 1018.6 |
| 16.7313 | 1108.92 |
| 16.8045 | 1118.86 |
| 16.4008 | 1109.91 |
| 16.8031 | 1134.63 |
| 16.6876 | 963.448 |
| 16.4787 | 1234.82 |
| 16.36 | 1066.8 |
| 16.3348 | 919.899 |
| 16.6911 | 980.444 |
| 16.6345 | 1076.47 |
| 16.7402 | 890.328 |
| 16.6105 | 930.007 |
| 16.9447 | 1089.06 |
| 16.3121 | 885.627 |
| 16.5764 | 952.384 |
| 16.6095 | 1058.24 |
| 16.6575 | 1025.04 |
| 16.3315 | 1072.75 |
| 16.6944 | 1010.29 |
| 16.9949 | 1033.21 |
| 16.4485 | 1064 |
| 17.1999 | 1049.55 |
| 16.6319 | 1056.33 |
| 16.7077 | 932.228 |

| Total Mean Effectiveness (QALYs) | Strategy 6 |
| --- | --- |
| 16.8249 | 1048.69 |
| 16.296 | 1332.43 |
| 16.9386 | 1094.91 |
| 16.6577 | 1151.95 |
| 16.4759 | 1134.3 |
| 16.3815 | 1249.05 |
| 16.4233 | 1055.6 |
| 16.7143 | 1057.92 |
| 16.7427 | 1242.26 |
| 16.8298 | 1053.96 |
| 16.3324 | 1107.9 |
| 16.5302 | 1088.33 |
| 16.7325 | 1067.46 |
| 16.8104 | 1187.21 |
| 16.1998 | 1202.25 |
| 16.7556 | 1084.46 |
| 16.8391 | 1283.73 |
| 16.4054 | 1056.15 |
| 16.7566 | 1282.14 |
| 16.4995 | 1152.03 |
| 16.309 | 1078.35 |
| 16.7855 | 1111.93 |
| 16.3226 | 1185.16 |
| 16.4034 | 1105.59 |
| 16.4001 | 1041.92 |
| 16.7124 | 1117.87 |
| 16.4019 | 1059.65 |
| 16.2278 | 1053.22 |
| 16.8716 | 1158.92 |
| 16.3696 | 1273.18 |
| 16.6697 | 1301.5 |
| 16.118 | 1169.75 |
| 16.7758 | 1038.85 |
| 16.8507 | 1141.79 |
| 17.0858 | 1270.86 |
| 16.5872 | 1219.9 |
| 16.84 | 1025.51 |
| 15.9519 | 1079.74 |
| 16.7638 | 1029.53 |
| 16.9779 | 1259.02 |
| 16.1105 | 1088.89 |
| 16.3883 | 1399.43 |
| 17.209 | 1113.88 |
| 16.6225 | 1075.19 |
| 16.7095 | 1075.74 |
| 16.5814 | 1094.7 |
| 16.8206 | 1199.07 |
| 16.8493 | 1120.15 |
| 16.5233 | 1243.83 |
| 16.28 | 1119.38 |
| 15.9207 | 1246.13 |
| 16.6057 | 1220.42 |
| 16.6764 | 1149.48 |
| 16.2397 | 1241.58 |
| 16.598 | 1201.08 |
| 16.73 | 1086.13 |
| 16.6721 | 1161.14 |
| 16.5743 | 1088.62 |
| 16.4939 | 1136.43 |
| 16.9986 | 1083.09 |
| 16.7699 | 1230.56 |
| 16.7603 | 1168.19 |
| 16.1709 | 1200.36 |
| 16.7949 | 964.723 |
| 17.142 | 1185.59 |
| 16.4333 | 1091.14 |
| 16.7859 | 1132.35 |
| 16.6532 | 1208.9 |
| 16.8772 | 1119.73 |
| 16.6907 | 1050.53 |
| 16.747 | 1166.1 |
| 16.5036 | 1012.8 |
| 16.2149 | 1124.15 |
| 16.1421 | 1198.94 |
| 17.0926 | 1213.77 |
| 16.4261 | 1076.62 |
| 16.2852 | 1100.7 |
| 16.6341 | 1134.43 |
| 17.2695 | 1050.06 |
| 16.6313 | 1054.94 |
| 17.0527 | 1192.94 |
| 16.878 | 993.523 |
| 16.5778 | 1212.97 |
| 16.9049 | 1244.59 |
| 16.5835 | 1199.32 |
| 16.3132 | 1144.49 |
| 16.8436 | 1044.23 |
| 16.6355 | 1105.85 |
| 16.937 | 1094.54 |
| 16.6636 | 1230.43 |
| 17.0197 | 987.777 |
| 16.4724 | 1048.66 |
| 16.7967 | 1162.51 |
| 16.7567 | 1222.35 |
| 16.4884 | 1258.41 |
| 16.7688 | 1124.57 |
| 16.8315 | 1143.57 |
| 16.7667 | 1205.93 |
| 16.7472 | 1005.11 |
| 16.3903 | 1153.7 |
| 16.8104 | 1217.62 |
| 16.5868 | 1242.88 |
| 16.3328 | 1184.65 |
| 16.7573 | 1122.16 |
| 16.7059 | 1083.13 |
| 17.1569 | 1168.25 |
| 16.5001 | 1179.77 |
| 16.3962 | 1217.09 |
| 17.1542 | 1204.5 |
| 16.207 | 1082.41 |
| 16.2032 | 1047.31 |
| 16.3258 | 1177.03 |
| 16.6656 | 1077.13 |
| 16.9793 | 1181.53 |
| 16.5367 | 946.855 |
| 16.5646 | 1213.54 |
| 16.8 | 1051.77 |
| 16.7469 | 1071.48 |
| 16.5409 | 1132.78 |
| 16.4635 | 1131.44 |
| 17.0755 | 1333.54 |
| 16.8532 | 1181.4 |
| 16.7779 | 1207.24 |
| 16.5203 | 1105.52 |
| 16.7912 | 1171.58 |
| 16.9413 | 1180.5 |
| 16.6436 | 1124.12 |
| 16.9079 | 1190.32 |
| 16.4117 | 1231.86 |
| 16.9125 | 1084.75 |
| 17.0544 | 1073.46 |
| 16.8359 | 1055.14 |
| 16.4305 | 1113.66 |
| 16.3155 | 1073.23 |
| 16.4452 | 1099.97 |
| 16.2971 | 1085.1 |
| 16.6541 | 1086.78 |
| 16.4393 | 1129.32 |
| 16.5542 | 1319.84 |
| 16.9599 | 1095.18 |
| 16.9591 | 1145.65 |
| 16.7821 | 1245.3 |
| 16.5578 | 1148.62 |
| 16.9758 | 1074.8 |
| 16.4998 | 1104.69 |
| 16.2017 | 1208.96 |
| 17.237 | 1284.7 |
| 16.772 | 1299.76 |
| 16.7986 | 1339.5 |
| 16.786 | 1274.53 |
| 16.6276 | 1106.5 |
| 16.6681 | 1071.55 |
| 16.7538 | 1154.85 |
| 16.366 | 1110.17 |
| 16.4399 | 1035.71 |
| 16.7269 | 1036.75 |
| 16.554 | 1396.05 |
| 16.949 | 999.498 |
| 16.9118 | 1152.37 |
| 16.6481 | 1159.67 |
| 16.3588 | 1235.47 |
| 16.4755 | 1220.81 |
| 16.6056 | 1286.01 |
| 16.833 | 1234.89 |
| 16.7282 | 1095.99 |
| 16.7655 | 1142.15 |
| 16.7436 | 1022.75 |
| 16.5259 | 1219.83 |
| 16.3344 | 1140.08 |
| 16.1401 | 1277.43 |
| 16.8317 | 1270.85 |
| 16.2837 | 1027.45 |
| 16.2565 | 1318.23 |
| 16.4776 | 1200.74 |
| 16.5255 | 1269.32 |
| 16.5027 | 1183.15 |
| 16.2397 | 1089.75 |
| 16.5706 | 1390.18 |
| 16.5487 | 1221.32 |
| 16.8266 | 1033.96 |
| 16.4812 | 1167.86 |
| 16.3521 | 1155.85 |
| 17.0017 | 1207.71 |
| 16.4828 | 1189.63 |
| 16.8012 | 1298.04 |
| 16.511 | 1142.64 |
| 16.7991 | 1092.77 |
| 16.8079 | 1071.14 |
| 16.6047 | 1060.83 |
| 16.6682 | 1135.45 |
| 17.0776 | 1275.28 |
| 16.5492 | 1092.13 |
| 16.695 | 1137.07 |
| 16.8431 | 1113.53 |
| 16.8622 | 1305.82 |
| 16.5845 | 1192.92 |
| 16.4351 | 1031.77 |
| 16.6472 | 1183.02 |
| 16.8591 | 1116.33 |
| 16.6125 | 1217.77 |

| Total Mean Effectiveness (QALYs) | Comparator |
| --- | --- |
| 16.7421 | 859.959 |
| 16.7974 | 1053.12 |
| 16.774 | 1054.41 |
| 16.398 | 932.91 |
| 16.373 | 839.048 |
| 16.5313 | 837.634 |
| 16.3757 | 1125.86 |
| 16.4886 | 859.404 |
| 16.4725 | 846.11 |
| 16.9172 | 845.304 |
| 16.3467 | 893.52 |
| 16.6737 | 898.7 |
| 17.114 | 825.573 |
| 16.5195 | 1010.51 |
| 16.5021 | 913.473 |
| 16.2463 | 793.793 |
| 16.4187 | 1029.66 |
| 16.4314 | 935.064 |
| 16.6151 | 1012.65 |
| 16.8501 | 849.582 |
| 16.6829 | 1019.96 |
| 16.88 | 837.466 |
| 16.7475 | 862.725 |
| 16.7564 | 894.417 |
| 16.8224 | 889.923 |
| 16.8838 | 888.068 |
| 16.6868 | 959.633 |
| 16.7767 | 948.062 |
| 16.5923 | 861.896 |
| 16.4944 | 898.214 |
| 16.6564 | 978.534 |
| 16.7026 | 1016.48 |
| 16.4411 | 861.438 |
| 16.9116 | 908.488 |
| 15.9371 | 875.835 |
| 16.3445 | 838.139 |
| 16.5055 | 794.144 |
| 16.547 | 894.171 |
| 16.4575 | 962.176 |
| 16.782 | 1009.45 |
| 17.303 | 799.689 |
| 16.6571 | 978.379 |
| 16.3354 | 960.313 |
| 16.6884 | 934.687 |
| 16.8931 | 922.618 |
| 16.757 | 922.711 |
| 16.2919 | 793.998 |
| 16.3372 | 918.358 |
| 16.4502 | 921.117 |
| 16.6896 | 953.619 |
| 16.506 | 868.18 |
| 16.7149 | 922.514 |
| 16.3553 | 1103.87 |
| 16.7887 | 1026.45 |
| 16.8216 | 920.8 |
| 16.3161 | 968.231 |
| 16.8488 | 810.014 |
| 16.7442 | 919.947 |
| 16.1998 | 836.515 |
| 16.7232 | 960.367 |
| 16.3872 | 1050.71 |
| 16.7202 | 921.616 |
| 16.4091 | 1093.11 |
| 16.7992 | 853.837 |
| 16.789 | 912.113 |
| 16.5877 | 987.571 |
| 16.9629 | 939.094 |
| 16.9934 | 864.155 |
| 17.1418 | 1022.68 |
| 17.1493 | 937.763 |
| 16.9334 | 916.929 |
| 16.4391 | 864.829 |
| 16.3589 | 1000.68 |
| 16.7254 | 766.679 |
| 16.8802 | 906.53 |
| 16.7916 | 894.863 |
| 17.0036 | 885.024 |
| 16.8307 | 955.492 |
| 16.7307 | 1030.89 |
| 16.5291 | 906.275 |
| 16.341 | 769.638 |
| 16.9145 | 931.807 |
| 16.7117 | 918.155 |
| 16.9747 | 842.682 |
| 16.6131 | 917.293 |
| 15.9932 | 910.848 |
| 16.5815 | 927.217 |
| 17.0314 | 945.615 |
| 16.5979 | 940.325 |
| 16.9687 | 967.209 |
| 16.2992 | 1006.11 |
| 16.7548 | 1011.66 |
| 16.8849 | 926.996 |
| 16.7648 | 913.222 |
| 16.893 | 875.627 |
| 16.5867 | 876.976 |
| 16.5748 | 894.086 |
| 16.4674 | 988.73 |
| 16.1875 | 857.32 |
| 16.7175 | 871.839 |
| 16.4337 | 840.636 |
| 16.5373 | 906.343 |
| 16.4814 | 834.313 |
| 16.8486 | 979.683 |
| 16.7168 | 920.723 |
| 16.4308 | 1176.64 |
| 16.8906 | 904.19 |
| 16.6193 | 814.352 |
| 16.5973 | 959.014 |
| 16.7414 | 909.72 |
| 16.9398 | 966.467 |
| 16.8209 | 934.37 |
| 16.5707 | 1039.88 |
| 17.0718 | 918.516 |
| 16.6995 | 992.995 |
| 17.0466 | 932.036 |
| 16.5523 | 976.261 |
| 16.5102 | 953.643 |
| 16.7335 | 904.919 |
| 16.759 | 913.991 |
| 16.2594 | 930.88 |
| 16.5819 | 1000.62 |
| 16.8515 | 814.433 |
| 16.5925 | 902.617 |
| 16.2663 | 928.986 |
| 16.8358 | 997.477 |
| 16.7351 | 922.923 |
| 16.8533 | 1084.7 |
| 16.761 | 905.722 |
| 16.1316 | 1025.87 |
| 16.4115 | 990.502 |
| 17.1072 | 895.035 |
| 16.5302 | 862.88 |
| 16.4299 | 909.84 |
| 16.5147 | 821.813 |
| 15.8478 | 947.207 |
| 16.4621 | 1136.84 |
| 16.2338 | 948.097 |
| 16.5674 | 896.586 |
| 16.358 | 861.782 |
| 16.2039 | 866.663 |
| 16.3437 | 876.103 |
| 16.6083 | 799.617 |
| 16.841 | 815.129 |
| 16.488 | 923.349 |
| 16.8556 | 994.447 |
| 16.8171 | 1036.4 |
| 16.7809 | 1029.32 |
| 17.0105 | 981.745 |
| 16.8685 | 1001.92 |
| 16.7433 | 868.841 |
| 16.7835 | 957.825 |
| 16.8391 | 968.407 |
| 16.5406 | 880.903 |
| 16.3243 | 872.253 |
| 16.3779 | 886.516 |
| 16.8157 | 851.06 |
| 16.962 | 802.357 |
| 16.5966 | 865.839 |
| 16.388 | 1040.46 |
| 16.7748 | 927.535 |
| 16.5794 | 857.243 |
| 16.3621 | 941.184 |
| 16.572 | 970.456 |
| 16.3915 | 796.314 |
| 16.7234 | 911.66 |
| 16.9386 | 1007.65 |
| 16.7435 | 862.329 |
| 16.391 | 810.863 |
| 16.8026 | 930.243 |
| 16.4737 | 805.958 |
| 16.7364 | 919.843 |
| 16.2635 | 997.794 |
| 17.0387 | 1007.7 |
| 16.7446 | 858.916 |
| 16.6302 | 1037.46 |
| 16.4955 | 1042.12 |
| 16.5533 | 879.491 |
| 16.4812 | 1003.97 |
| 16.3467 | 855.57 |
| 16.3676 | 944.948 |
| 16.8563 | 930.158 |
| 16.5954 | 858.658 |
| 16.9996 | 990.715 |
| 16.8318 | 901.723 |
| 16.6435 | 1009.3 |
| 16.9563 | 934.414 |
| 16.6201 | 1098.04 |
| 16.7232 | 834.812 |
| 16.8675 | 815.293 |
| 16.7339 | 863.443 |
| 16.893 | 879.553 |
| 16.7225 | 979.895 |
| 16.4082 | 1059.79 |
| 16.8453 | 985.366 |
| 16.6602 | 1029.2 |
| 16.9836 | 1010.38 |
| 16.6128 | 897.978 |
| 16.067 | 918.092 |
| 16.1989 | 969.623 |

**Figure (2). Cost-Effectiveness Plane ( PSA:1000 simulations)**

Comparator (current situation) 40-70, annual attendance rate 3.5%, Strategy1 40-60, biennial, attendance rate 50%, Strategy2 50-65, annual, attendance rate 50%, Strategy3 50-65, biennial, attendance rate 50%, Strategy4 40-70, biennial, attendance rate 50%, Strategy5, 50-70 biennial, attendance rate 50%, Strategy6 40-49 annual, 50-70 biennial, attendance rate 50%. PSA: Probablistic Sensistivity Analysis

Probability of being Cost Effective

| Cost Effectiveness Threshold US$/QALY | Comparator | Strategy 1 | Strategy 2 | Strategy 3 | Strategy 4 | Strategy 5 | Strategy 6 |
| --- | --- | --- | --- | --- | --- | --- | --- |
| 0 | 0.67 | 0.025 | 0.008 | 0.172 | 0.007 | 0.108 | 0.01 |
| 1000 | 0.296 | 0.109 | 0.085 | 0.177 | 0.098 | 0.16 | 0.075 |
| 2000 | 0.225 | 0.128 | 0.105 | 0.158 | 0.135 | 0.149 | 0.1 |
| 3000 | 0.192 | 0.133 | 0.118 | 0.157 | 0.147 | 0.145 | 0.108 |
| 4000 | 0.18 | 0.135 | 0.123 | 0.156 | 0.151 | 0.14 | 0.115 |
| 5000 | 0.168 | 0.138 | 0.127 | 0.156 | 0.157 | 0.139 | 0.115 |
| 6000 | 0.166 | 0.138 | 0.129 | 0.152 | 0.159 | 0.138 | 0.118 |
| 7000 | 0.159 | 0.139 | 0.128 | 0.155 | 0.161 | 0.137 | 0.121 |
| 8000 | 0.157 | 0.141 | 0.129 | 0.151 | 0.163 | 0.135 | 0.124 |
| 9000 | 0.155 | 0.141 | 0.131 | 0.151 | 0.164 | 0.135 | 0.123 |
| 10000 | 0.153 | 0.142 | 0.131 | 0.15 | 0.165 | 0.135 | 0.124 |
| 11000 | 0.153 | 0.141 | 0.131 | 0.149 | 0.166 | 0.135 | 0.125 |
| 12000 | 0.152 | 0.142 | 0.131 | 0.149 | 0.168 | 0.134 | 0.124 |
| 13000 | 0.151 | 0.143 | 0.131 | 0.148 | 0.169 | 0.134 | 0.124 |
| 14000 | 0.151 | 0.143 | 0.131 | 0.147 | 0.169 | 0.134 | 0.125 |
| 15000 | 0.149 | 0.144 | 0.131 | 0.146 | 0.17 | 0.134 | 0.126 |
| 16000 | 0.147 | 0.145 | 0.131 | 0.145 | 0.171 | 0.134 | 0.127 |
| 17000 | 0.146 | 0.145 | 0.131 | 0.145 | 0.171 | 0.134 | 0.128 |
| 18000 | 0.146 | 0.145 | 0.13 | 0.145 | 0.171 | 0.135 | 0.128 |
| 19000 | 0.146 | 0.144 | 0.13 | 0.145 | 0.172 | 0.135 | 0.128 |
| 20000 | 0.145 | 0.144 | 0.131 | 0.145 | 0.172 | 0.135 | 0.128 |
| 21000 | 0.145 | 0.144 | 0.131 | 0.145 | 0.173 | 0.134 | 0.128 |
| 22000 | 0.145 | 0.144 | 0.131 | 0.145 | 0.173 | 0.134 | 0.128 |
| 23000 | 0.145 | 0.144 | 0.131 | 0.145 | 0.173 | 0.134 | 0.128 |
| 24000 | 0.145 | 0.144 | 0.131 | 0.145 | 0.173 | 0.134 | 0.128 |
| 25000 | 0.145 | 0.144 | 0.131 | 0.145 | 0.173 | 0.134 | 0.128 |
| 26000 | 0.145 | 0.144 | 0.131 | 0.145 | 0.173 | 0.134 | 0.128 |
| 27000 | 0.144 | 0.145 | 0.131 | 0.145 | 0.173 | 0.134 | 0.128 |
| 28000 | 0.144 | 0.145 | 0.131 | 0.145 | 0.173 | 0.134 | 0.128 |
| 29000 | 0.143 | 0.145 | 0.131 | 0.145 | 0.173 | 0.135 | 0.128 |
| 30000 | 0.143 | 0.145 | 0.131 | 0.145 | 0.173 | 0.135 | 0.128 |
| 31000 | 0.142 | 0.145 | 0.131 | 0.145 | 0.173 | 0.135 | 0.129 |
| 32000 | 0.142 | 0.145 | 0.131 | 0.145 | 0.173 | 0.135 | 0.129 |
| 33000 | 0.142 | 0.145 | 0.131 | 0.145 | 0.173 | 0.135 | 0.129 |
| 34000 | 0.142 | 0.145 | 0.131 | 0.145 | 0.173 | 0.135 | 0.129 |
| 35000 | 0.142 | 0.145 | 0.131 | 0.145 | 0.173 | 0.135 | 0.129 |
| 36000 | 0.142 | 0.145 | 0.131 | 0.145 | 0.173 | 0.135 | 0.129 |
| 37000 | 0.142 | 0.145 | 0.131 | 0.145 | 0.173 | 0.135 | 0.129 |
| 38000 | 0.142 | 0.145 | 0.131 | 0.145 | 0.173 | 0.135 | 0.129 |
| 39000 | 0.142 | 0.145 | 0.131 | 0.145 | 0.173 | 0.135 | 0.129 |
| 40000 | 0.142 | 0.145 | 0.131 | 0.145 | 0.173 | 0.135 | 0.129 |
| 41000 | 0.142 | 0.145 | 0.131 | 0.145 | 0.173 | 0.135 | 0.129 |
| 42000 | 0.142 | 0.145 | 0.131 | 0.145 | 0.173 | 0.135 | 0.129 |
| 43000 | 0.142 | 0.145 | 0.131 | 0.145 | 0.173 | 0.135 | 0.129 |
| 44000 | 0.142 | 0.145 | 0.131 | 0.145 | 0.173 | 0.135 | 0.129 |
| 45000 | 0.142 | 0.145 | 0.131 | 0.145 | 0.173 | 0.135 | 0.129 |
| 46000 | 0.142 | 0.145 | 0.131 | 0.144 | 0.173 | 0.136 | 0.129 |
| 47000 | 0.142 | 0.145 | 0.131 | 0.144 | 0.173 | 0.136 | 0.129 |
| 48000 | 0.142 | 0.145 | 0.131 | 0.144 | 0.173 | 0.136 | 0.129 |
| 49000 | 0.142 | 0.145 | 0.131 | 0.144 | 0.173 | 0.136 | 0.129 |
| 50000 | 0.142 | 0.145 | 0.131 | 0.144 | 0.173 | 0.136 | 0.129 |
| 51000 | 0.142 | 0.145 | 0.131 | 0.144 | 0.173 | 0.136 | 0.129 |
| 52000 | 0.142 | 0.145 | 0.131 | 0.144 | 0.173 | 0.136 | 0.129 |
| 53000 | 0.142 | 0.145 | 0.131 | 0.144 | 0.173 | 0.136 | 0.129 |
| 54000 | 0.142 | 0.145 | 0.131 | 0.144 | 0.173 | 0.136 | 0.129 |
| 55000 | 0.142 | 0.145 | 0.131 | 0.144 | 0.173 | 0.136 | 0.129 |

**Figure (3). Cost-Effectiveness Acceptability Curve**

Comparator (current situation) 40-70, annual attendance rate 3.5%, Strategy1 40-60, biennial, attendance rate 50%, Strategy2 50-65, annual, attendance rate 50%, Strategy3 50-65, biennial, attendance rate 50%, Strategy4 40-70, biennial, attendance rate 50%, Strategy5, 50-70 biennial, attendance rate 50%, Strategy6 40-49 annual, 50-70 biennial, attendance rate 50%.

Total Mean cost US$

| Total Mean QALYs | Comparator |
| --- | --- |
| 16.9802 | 879.719 |

| Total Mean QALYs | Scenario_3 |
| --- | --- |
| 16.9926 | 963.533 |

| Total Mean QALYs | Scenario_5 |
| --- | --- |
| 16.9929 | 993.86 |

| Total Mean QALYs | Scenario_1 |
| --- | --- |
| 16.9963 | 1078.3 |

| Total Mean QALYs | Scenario_2 |
| --- | --- |
| 16.9978 | 1102.29 |

| Total Mean QALYs | Scenario_4 |
| --- | --- |
| 17.002 | 1132.85 |

| Total Mean QALYs | Scenario_6 |
| --- | --- |
| 17.0043 | 1274.89 |

**Figure(4). Cost-Effectiveness Frontier**

Comparator (current situation) 40-70, annual attendance rate 3.5%, Strategy1 40-60, biennial, attendance rate 50%, Strategy2 50-65, annual, attendance rate 50%, Strategy3 50-65, biennial, attendance rate 50%, Strategy4 40-70, biennial, attendance rate 50%, Strategy5, 50-70 biennial, attendance rate 50%, Strategy6 40-49 annual, 50-70 biennial, attendance rate 50%. This frontier is based on the model’s deterministic results, and the frontier line shows the most efficient screening strategies

Probability of being Cost Effective

| Cost Effectiveness Threshold US$/QALY | Comparator | Strategy 4 |
| --- | --- | --- |
| 0 | 0.67 |  |
| 1000 | 0.296 |  |
| 2000 | 0.225 |  |
| 3000 | 0.192 |  |
| 4000 | 0.18 |  |
| 5000 | 0.168 |  |
| 6000 | 0.166 |  |
| 7000 |  | 0.161 |
| 8000 |  | 0.163 |
| 9000 |  | 0.164 |
| 10000 |  | 0.165 |
| 11000 |  | 0.166 |
| 12000 |  | 0.168 |
| 13000 |  | 0.169 |
| 14000 |  | 0.169 |
| 15000 |  | 0.17 |
| 16000 |  | 0.171 |
| 17000 |  | 0.171 |
| 18000 |  | 0.171 |
| 19000 |  | 0.172 |
| 20000 |  | 0.172 |
| 21000 |  | 0.173 |
| 22000 |  | 0.173 |
| 23000 |  | 0.173 |
| 24000 |  | 0.173 |
| 25000 |  | 0.173 |
| 26000 |  | 0.173 |
| 27000 |  | 0.173 |
| 28000 |  | 0.173 |
| 29000 |  | 0.173 |
| 30000 |  | 0.173 |
| 31000 |  | 0.173 |
| 32000 |  | 0.173 |
| 33000 |  | 0.173 |
| 34000 |  | 0.173 |
| 35000 |  | 0.173 |
| 36000 |  | 0.173 |
| 37000 |  | 0.173 |
| 38000 |  | 0.173 |
| 39000 |  | 0.173 |
| 40000 |  | 0.173 |
| 41000 |  | 0.173 |
| 42000 |  | 0.173 |
| 43000 |  | 0.173 |
| 44000 |  | 0.173 |
| 45000 |  | 0.173 |
| 46000 |  | 0.173 |
| 47000 |  | 0.173 |
| 48000 |  | 0.173 |
| 49000 |  | 0.173 |
| 50000 |  | 0.173 |
| 51000 |  | 0.173 |
| 52000 |  | 0.173 |
| 53000 |  | 0.173 |
| 54000 |  | 0.173 |
| 55000 |  | 0.173 |

**Figure(5). Cost-Effectiveness Acceptability Frontier**

Comparator (current situation): 40-70, annual attendance rate 3.5%, Strategy4: 40-70, biennial, attendance rate 50%

**References**

1.	de Gelder R, Heijnsdijk EA, Fracheboud J, Draisma G, de Koning HJ. The effects of population-based mammography screening starting between age 40 and 50 in the presence of adjuvant systemic therapy. Int J Cancer. 2015;137(1):165-72.

2.	Abdel-Razeq H, Mansour A, Jaddan D. Breast Cancer Care in Jordan. JCO Glob Oncol. 2020;6:260-8.

3.	Schiller-Fruehwirth I, Jahn B, Einzinger P, Zauner G, Urach C, Siebert U. The Long-Term Effectiveness and Cost Effectiveness of Organized versus Opportunistic Screening for Breast Cancer in Austria. Value Health. 2017;20(8):1048-57.

4.	Ajlouni R. Jordan Breast Cancer Program Attendance Rate. 2022.

5.	Al Rabayah A, Roudijk B, Purba FD, Rencz F, Jaddoua S, Siebert U. Valuation of the EQ-5D-3L in Jordan. Eur J Health Econ. 2025;26(3):487-501.

6.	Kaur MN, Yan J, Klassen AF, David JP, Pieris D, Sharma M, et al. A Systematic Literature Review of Health Utility Values in Breast Cancer. Med Decis Making. 2022;42(5):704-19.

7.	King Hussein Cancer Center. Cancer Cost per Stage KHCC dashborad 2023.

8.	Mousa R, Hammad E, Melhem J, Al-Jaghbir M. Direct medical costs of breast cancer in Jordan: cost drivers and predictors. Expert Rev Pharmacoecon Outcomes Res. 2021;21(4):647-54.

9.	Alefan Q, Saadeh A, Yaghan RJ. Direct medical costs for stage-specific breast cancer: a retrospective analysis. Breast Cancer Management. 2020;9(1):BMT33.
